# Three-dimensional Imaging Reveals Immune-driven Tumor-associated High Endothelial Venules as a Key Correlate of Tumor Rejection Following Depletion of Regulatory T Cells

**DOI:** 10.1158/2767-9764.CRC-21-0123

**Published:** 2022-12-15

**Authors:** Stefan Milutinovic, Jun Abe, Emma Jones, Inken Kelch, Kathryn Smart, Sarah N. Lauder, Michelle Somerville, Carl Ware, Andrew Godkin, Jens V. Stein, Gib Bogle, Awen Gallimore

**Affiliations:** 1 Systems Immunity University Research Institute, Henry Wellcome Building, School of Medicine, Cardiff University, Cardiff, United Kingdom; 2 Department of Oncology, Microbiology and Immunology, University of Fribourg, Fribourg, Switzerland; 3 School of Biological Sciences, University of Auckland, Auckland, New Zealand; 4 Laboratory of Molecular Immunology, Sanford Burnham Prebys, La Jolla, California; 5 Auckland Bioengineering Institute, University of Auckland, Auckland, New Zealand

## Abstract

High endothelial venules (HEV) are specialized post capillary venules that recruit naïve T cells and B cells into secondary lymphoid organs (SLO) such as lymph nodes (LN). Expansion of HEV networks in SLOs occurs following immune activation to support development of an effective immune response. In this study, we used a carcinogen-induced model of fibrosarcoma to examine HEV remodeling after depletion of regulatory T cells (Treg). We used light sheet fluorescence microscopy imaging to visualize entire HEV networks, subsequently applying computational tools to enable topological mapping and extraction of numerical descriptors of the networks. While these analyses revealed profound cancer- and immune-driven alterations to HEV networks within LNs, these changes did not identify successful responses to treatment. The presence of HEV networks within tumors did however clearly distinguish responders from nonresponders. Finally, we show that a successful treatment response is dependent on coupling tumor-associated HEV (TA-HEV) development to T-cell activation implying that T-cell activation acts as the trigger for development of TA-HEVs which subsequently serve to amplify the immune response by facilitating extravasation of T cells into the tumor mass.

## Introduction

High endothelial venules (HEV) are specialized blood vessels, which are usually only found in secondary lymph organs where they enable recruitment of L-selectin–expressing lymphocytes into the lymph node (LN) parenchyma where they may encounter cognate antigen (1, 2). It is well known that HEV networks exhibit functional and structural changes in response to antigen stimulation accompanied by extensive LN enlargement (3, 4). Under these conditions of immune activation, HEV networks increase in length and branching, reflecting endothelial cell proliferation and the co-operative activities of vascular, stromal, or mesenchymal (fibroblastic reticular cells) and immune cells; most notably dendritic cells, T cells, and B cells (5–8). Such changes are associated with an increase in blood flow and lymphocyte trafficking to support the development of an adaptive immune response (3, 4, 9).

There is evidence that the immune system can recognize and eliminate newly transformed cells implying that immune activation occurs during the early stages of tumor development (10–14). Cancer cells however evolve immune evasion strategies that may be cancer cell intrinsic or extrinsic; in the case of the latter, cancer-driven immunosuppressive mechanisms are deployed to disable the immune system leading to cancer outgrowth. Conversely, when immunosuppressive mechanisms are targeted by immunotherapeutic interventions such as checkpoint inhibitors (15–17) or depletion of regulatory T cells (Treg; refs. 18, 19), the immune system is unleashed, sometimes resulting in control of tumor growth. While expansion of HEV networks has been characterized in the context of immune activation by foreign antigens (5–8, 20), such changes have not been similarly analyzed in the context of tumors.

An increasing number of reports indicate that vessels with characteristics of HEV [most notably expression of peripheral node addressin (PNAd)] are often found in tumors (reviewed in refs. 21, 22). The first large-scale studies of HEVs in primary human solid tumors and its association with lymphocyte infiltration were first reported by Martinet and colleagues (23). In addition to being associated with control of tumor growth, the expression of both naïve T cell and Th1 genes correlated with HEV density in human melanoma further supporting the role of tumor-associated HEVs (TA-HEV) as active sites of lymphocyte recruitment and activation (24). TA-HEVs are frequently found inside tertiary lymphoid structures (25–27) and are associated with a favorable response to cancer immunotherapy (28). In our own studies using a mouse model of carcinogen [3-methylcholanthrene (MCA)]-induced fibrosarcomas (18, 19, 29), we found that depleting Foxp3^+^ Tregs resulted in the development of PNAd^+^ vessels in some tumors where they are associated with a higher number of tumor-infiltrating lymphocytes (TIL) and control of tumor growth.

The MCA carcinogenesis model described above has been extensively used to explore the concept of cancer immunosurveillance and antitumor immunity. Several studies have demonstrated an increased susceptibility to MCA-induced sarcomas using mice deficient in either IFNγ or perforin as well as those which lack natural killer T cells, T cells, and B cells (10–12, 30–32). Furthermore, mice with limited or no T-cell receptor diversity were recently shown to have increased tumor risk further implicating T cells in the immune-mediated control of tumors (33). With these findings in mind, we selected this tumor model to examine the impact of tumors on HEV networks. We sought to test the hypothesis that specific changes to LN and TA-HEV networks underpin successful responses to Tregdepletion. To achieve this goal, we sought to move away from staining and analyzing thin tissue sections and instead to comprehensively map HEV networks by three-dimensional (3D) imaging. It is well recognized that tumors exhibit substantial heterogeneity and that capturing this feature leads to more accurate tumor profiling and staging (34–37). By applying such techniques, we explored different methods of inducing HEV in tumors, specifically examining the impact of HEV induction on T-cell infiltration and control of tumor growth.

## Materials and Methods

### Mice and Induction of Tumors

All animal experiments were performed according to Home Office UK regulations. Foxp3^DTR^ mice and *Rag2*^−/−^ mice ages 8 to 15 weeks were injected subcutaneously into the left hind leg with 400 μg of MCA (Sigma-Aldrich) in 100 μL of olive oil under general anesthetic as described previously (19, 38). Mice were then monitored for tumor development weekly for up to 8 months. Tumor-bearing mice were culled before their tumors reached 1.5 cm in diameter or if tumors caused apparent discomfort.

Tumor size and growth were monitored every other day using calipers. Measurements of the tumor leg width, nontumor leg width, tumor width, and tumor height were recorded in millimetres. Subsequently, the tumor growth rate (k, days^−1^) was derived by taking the difference between the tumor and non-tumour leg diameters (*X*,mm) and using the equation for exponential growth: *Y* = *Y0 x exp* (*k x X*), which was performed in the statistical package Prism 7 (Graphpad).

The tumor cell line 4T1, obtained from ATCC in 2014 (CRL-2539, RRID: CVCL_0125) authenticated in the past year, was cultured for 10 to 14 days before being injected subcutaneously into the mammary fat pad of Female BALB/C mice. The cell line was authenticated using short tandem repeat analysis and processed using the ABI Prism 3500xl Genetic Analyzer. Mice were injected with 1 × 10^5^ 4T1 cells in total and tumor measurements taken following day 7 for up to three times a week as described previously (39). Tumor volume was determined using the equation: (length × width × short) × (3.14/6; where short equals the lower of the length and width measurements and provides an estimate of height). All experimental procedures were reviewed by Cardiff University Animal Welfare & Ethical Review Board and were conducted in accordance with UK Home Office Guidelines (PPL: PP763775).

### *In Vivo* Treatments

Following the detection of a palpable tumor, 0.3 μg of diphtheria toxin (DT, Native Antigen) dilutedin 100 μL of PBS (Gibco Life Technologies) was administered intraperitoneally every other day for at least three treatments to deplete Tregs *in vivo*. The anti-LTβR (lymphotoxin β receptor) agonist antibody, 4H8 (40) was injected intraperitoneally into Foxp3^DTR^ mice as 100 μg diluted in 100 μL of sterile PBS every 3 days for a total of 14days. To deplete T cells *in vivo*, 100 μg of anti-CD4 (Bio X cell, clone GK1.5, catalog no. BP0003-1) and anti-CD8 (Bio X cell, clone YTS 169.4, catalog no. BP0117) antibodies were administered every other day intraperitoneally beginning 1 day prior to DT as described previously (18).

BALB/C mice bearing 4T1 tumors were treated with the PI3Kδ inhibitor (PI-3065,AChemBlock) by daily oral gavage at 75mg/kg and vehicle-treated mice were given the equivalent volume of carrier solution (39).

### Immunofluorescence and IHC Staining and Microscopy

A total of 5-μm-thick tumor formalin-fixed paraffin-embedded (FFPE) sections were baked at 60°C for 45 minutes. Baked sections were then dewaxed in xylene and rehydrated in descending concentrations of ethanol before being placed under running tap water for 5 minutes. After a brief wash in distilled water, the sections were placed in Tris-Ethylenediaminetetraacetic acid (Tris-EDTA) antigen retrieval buffer (10 mmol/L Tris Base, 1 mmol/L EDTA, 0.05% Tween 20, pH 8.0) and heated for 20 minutes inside a pressure cooker. The slides were left to cool down for 30 minutes before being washed in PBS and blocked with 2.5% normal horse serum. Slides were then incubated in primary antibody overnight at 4°C, washed with PBS, incubated in secondary antibody, washed again, counterstained with Hoechst (14533, Sigma), and then mounted in ProLong Gold Antifade mountant (P36930, Thermo Fisher Scientific). The slides were then imaged using a Zeiss slide scanner (Zeiss Axioscan Z.1). Primary antibodies used included MECA-79 IgM (BD Biosciences, catalog no. 553863, RRID:AB_395099) 2.5 μg/mL, anti-CD3 (GA50361-2, Agilent) 2 μg/mL, and anti-B220 (BioLegend, catalog no. 103201, RRID:AB_312986) 0.25 μg/mL.

For IHC staining and quantification of LN HEVs, 5-μm-thick draining and nondraining LN FFPE sections were dewaxed, rehydrated, and antigen retrieved using Tris-EDTA as described above. The slides were blocked for 10 minutes in 0.3% hydrogen peroxide/PBS to block endogenous peroxidase activity. Nonspecific antibody binding was blocked by incubating slides in 2.5% horse serum for 30 minutes at room temperature. Slides were then incubated overnight at 4°C with 250 μL of primary antibody diluent prepared in 1% BSA/PBS. The following day the sections were washed 3 × 3 minutes in PBS and incubated with the relevant ImmPRESS horseradish peroxidase (HRP) polymer detection kit (VectorLabs) for 30 minutes. Slides were then washed 3 × 3 minutes in PBS before being incubated with the HRP Impact DAB (Vectorlabs, brown) for 3 minutes. After a brief wash in distilled water, slides were counterstained in Mayer Hematoxylin for 4.5 minutes before being incubated in bluing reagent (Sigma) for 1 minute to detect nuclei. To detect red blood cells, counterstained slides were incubated in alcoholic Eosin for 20 seconds before being dehydrated in 100% ethanol followed by xylene. Slides were mounted in DPX and covered with glass coverslips. Slides were left to dry overnight in a fume hood before being imaged with a Zeiss slide scanner (Zeiss Axioscan Z.1).

### Two-dimensional HEV Quantification

FFPE LN sections were stained with the MECA-79 IgM antibody and imaged using a Zeiss slide scanner. Measurement tools within the Zen software (blue edition) allowed for the manual measurement of individual PNAd^+^ vessels across whole LN sections to derive the HEV area in square microns (μm^2^). Subsequently several numerical parameters pertaining to HEV formation could be derived as described previously (41). This included the total HEV area (expressed as a percentage of the total LN area), HEV density [number of HEV vessels per unit area (1 mm^2^)of LN]and average HEV area.

### *In Vivo* HEV Labeling, Embedding, and Optical Clearing

MECA-79 IgM conjugation was performed according to vendor instructions using the Alexa Fluor (AF)-594 conjugation kit (Thermo Fisher Scientific, A20185). To remove toxic sodium azide, MECA-79 AF-594 was buffer exchanged with PBS twice by spinning at 12G for 10 minutes using Vivaspin 500 protein concentrators (GE healthcare). To label HEVs, tumor-bearing mice were placed in a warming incubator set at 37°C for 30 minutes to dilate blood vessels. Mice were then injected intravenously into the tail vein with 20 μg of MECA-79 AF-594 to label LN HEVs and 50μg MECA-79 AF-594 to label intratumoral HEVs. A total of 12–15 minutes postinjection mice were culled and the dLN (inguinal), ndLN (inguinal, opposite leg) and tumor were excised and fixed in 4% paraformaldehyde at 4°C overnight.

To reduce endogenous autofluorescence, fixed labeled tissues were washed in PBS for 30 minutes before being stepwise dehydrated in increasing concentrations of methanol (33%, 66%, and 100% methanol) for 15 minutes each as described previously (42). This was done to preserve the specimen for subsequent bleaching. Specimens were then incubated in modified Dent bleach (2:1:3 ratio of methanol, DMSO and 30% hydrogen peroxide) for 24 hours which lead to tissue decolourization, improved optical clearing and an enhanced signal to noise ratio.

Fixed labeled and bleached LNs were then embedded in 1.5% low melting point agarose (Invitrogen). Agarose blocks were cut into a cuboidal shape with the following dimensions: 8 mm × 8 mm × 25 mm as described previously (43). Agarose-embedded specimens were dehydrated in 100% methanol for 3 days (methanol replenished every 24 hours) to ensure complete removal of extracellular water. Specimens were then transferred to 60 mL of the organic clearing solvent benzyl alcohol and benzyl benzoate (BABB, 1:2 ratio) until complete transparency was achieved.

### Light Sheet Fluorescence Imaging and LN HEV Network Quantification

The precise Light sheet fluorescence microscopy (LSFM) instrument used to image optically cleared samples is specified in each individual figure legend. An implementation of LSFM termed selective plane illumination microscopy (SPIM) was used to image cleared agarose embedded specimens as described previously (43).All images were recorded using an N-PLAN EPI 5 × /0.12 objective and a 12-bit digital CCD camera. Multi-channel 3D stacks obtained at overlapping fields of view (FOV) were exported in the tagged image file format (TIFF) format and stitched using BigStitcher plugin for ImageJ (44). Stitched image files were rendered and viewed as maximum intensity projections (MIP) using IMARIS software v 9.2.1.

To generate global 3D topological descriptors of LN HEV networks acquired by SPIM imaging, a set of command line computational tools was implemented and further refined to process SPIM acquired datasets. These tools are freely accessible (https://github.com/gibbogle/vessel-tools) and have been described previously (45). The generated spatial graph files, which allow for the rending of the 3D LN HEV network, were visualized in Amira v 6.7.0.

### LSFM Imaging of Whole Tumors

The LaVision Ultramicroscope 2 (Miltenyi Biotec) was used to image intratumoral HEVs in whole tumors that had a maximum diameter of 6 mm. Cleared tumor samples were fixed in position using a screw mounting cap. The sample was lowered into the imaging chamber which was filled with 250 mL of ethyl cinnamate (ECi) which is a nontoxic organic solvent that has a similar refractive index to BABB (46). All images were recorded using a 2× detection objective with a working distance of 6 mm and a zoom ratio of 0.63× −6.3× (1:10). Depending on the size of the tumor, a magnification between 0.63× and 1.25× was used for a global overview of intratumoral HEVs and 2× for regions of interest with dense clusters of PNAd^+^ vessels. The z-step size used was 5 μm (at 0.63×−1.25×)and 2 μm (at 2×) with a light sheet width NA of 0.103. Multi-channel 3D stacks obtained at overlapping FOV were exported in the TIFF format and stitched using BigStitcher plugin (44).

### Statistical Analyses

All statistical analyses were performed using Prism 7.0 (GraphPad). The distribution of each dataset was determined using D’Agostino and Pearson omnibus normality test. Parametric analyses using one-way ANOVA with Tukey multiple comparison test was used for normally distributed datasets.For datasets that were not normally distributed, nonparametric analyses using Kruskal–Wallis test with Dunn multiple comparison test were performed.

## Results

### Fibrosarcoma Establishment Drives Extensive LN HEV Remodeling

We first examined the impact of fibrosarcoma establishment on HEV networks in draining (dLN) and nondraining (ndLN) inguinal LNs by bright-field microscopy imaging of sections stained with PNAd-specific antibodies. While fibrosarcoma-driven lymphadenopathy was observed, no differences in HEV area, density or distribution between dLNs and ndLNs could be detected using these methods (Supplementary Fig. S1). Because the observed disparity between lymphadenopathy and a failure to observe any measurable differences in the HEV networks suggested that the snapshots offered by imaging tissue sections were failing to capture the complexity of LN HEV, we applied a 3D LSFM approach to enable visualization of the entire HEV network.

**Figure 1:**
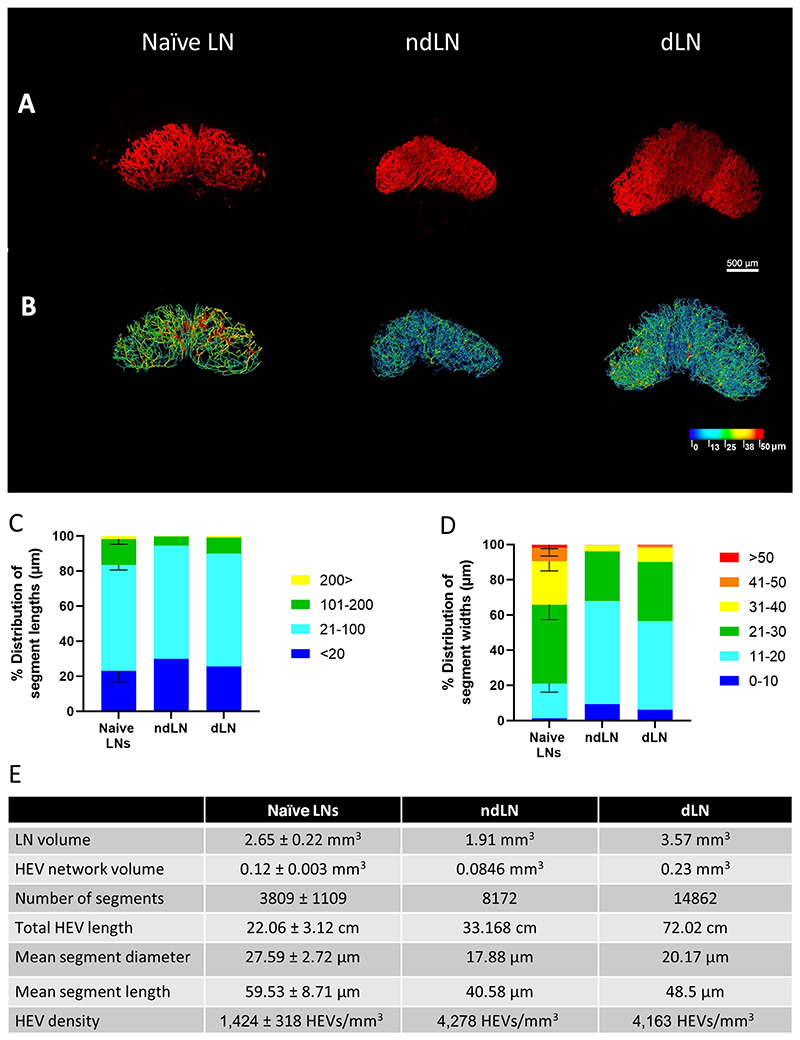
SPIM imaging and 3D analysis of LN HEV remodeling following fibrosarcoma establishment. Whole LNs were labeled *in vivo* with 20 μg MECA-79 594, cleared using a 1:2 ratio of BABB and imaged by SPIM. **A,** Representative MIPs of a tumor naïve inguinal LN (naïve LN), a fibrosarcoma ndLN (ndLN), and a fibrosarcoma dLN (dLN). **B,** Complementary 3D rendered models of LN HEV networks derived using the vessel tools are color coded to represent the vessel widths. Relative distribution of HEV segment lengths (**C**) and widths (**D**) of pooled naïve inguinal LNs (*n* = 3), ndLN (*n* = 1), and dLN (*n* = 1). Lengths and widths were divided into discrete bin (units shown are in μm) and the percentage of segments found in each bin was calculated and graphed. For pooled samples, the mean is displayed with error bars representing SDs from the mean. **E,** Extracted numerical parameters of LN HEVs.

To achieve this goal, mice were injected with fluorescently labeled PNAd-specific antibodies and LNs were removed, cleared in the organic solvent BABB and imaged by LSFM (Fig. 1A). To derive numerical descriptors of HEV networks, a set of computational tools originally developed for quantifying LN blood vessel networks (45) was further refined to identify discrete HEV networks which reflect the hierarchy of HEV branching structures within LN HEV networks (Supplementary Fig. S2A–S2C; ref. 47). The vessel tools were first validated by characterizing the 3D topology of popliteal and inguinal LN HEVs derived from tumor naïve mice (Supplementary Fig. S2D–S2F). A linear relationship between LN volume and total HEV length was found for both inguinal and popliteal LN HEVs (Supplementary Fig. S2G). Importantly, the total number of branch points was found to correlate with total HEV length (Supplementary Fig. S2H) suggesting comparable network branching structure between LNs of different sizes. These findings are in close agreement to those reported by previous studies (20, 48), corroborating the validity of our approach for globally mapping LN HEV network remodeling.

Next, fibrosarcoma dLN and ndLN were *in vivo* labeled, imaged, and analyzed using the vessel tool pipeline. As shown in Fig. 1A and B, a more extensive HEV network was observed in both the dLN and ndLN compared with naïve LNs indicating that the establishment of a tumor drives systemic LN HEV remodeling. This was confirmed by comparing the complexity of the individual LN HEV networks (Fig. 1E). In the presence of tumor, LN HEVs exhibited a more extensive network characterized by an increased number of segments and branching points as well as larger total HEV lengths. Interestingly while the segment length distributions were largely unaltered between naïve LN, ndLN, and dLN, the establishment of a tumor led to an alteration of the overall segment width distribution in the ndLN and dLN with most HEVs consisting of small 11–20 μm width sized vessels (Fig. 1C and D). Although vessel constriction and vessel stretching may lead to a reduction in width as described previously (20), a lack of any obvious changes to the vessel length distribution suggest that the smaller width vessels observed herein are most likely newly formed vessels. Indeed, several vessels of smaller widths can be seen in the ndLN and dLN 3D rendered HEV network model (Fig. 1B). Increases in HEV density and number of segments further support this notion. Such changes are compatible with an increase in the cellularity of ndLN and dLN, consistently observed in tumor-bearing mice (41, 49).

### Treg Depletion Drives Extensive LN HEV Remodeling

While the above data indicate that clear changes in LN HEV networks are observed in mice bearing established tumors, these clearly do not reflect induction of an immune response capable of inhibiting tumor progression. Our own studies have revealed that Treg are highly enriched in tumors and tumor dLNs of fibrosarcoma-bearing mice which serve to limit effective antitumor responses (18, 19, 29). Betts and colleagues showed that administration of the CD25-specific antibody, PC61, limited development of MCA-induced tumors. Later studies using Foxp3^DTR^ transgenic mice (50), to selectively deplete Tregs in fibrosarcoma-bearing mice, showed that tumor progression was controlled in a proportion of treated mice (responder mice; refs. 19, 51). Importantly, control of tumor growth in responders was mediated by CD8^+^Tcells (18, 19). We sought to examine the impact of LN HEV remodeling in response to Treg depletion with view to exploring whether alterations to HEV networks corresponded with immune-mediated control of tumor growth.

**Figure 2:**
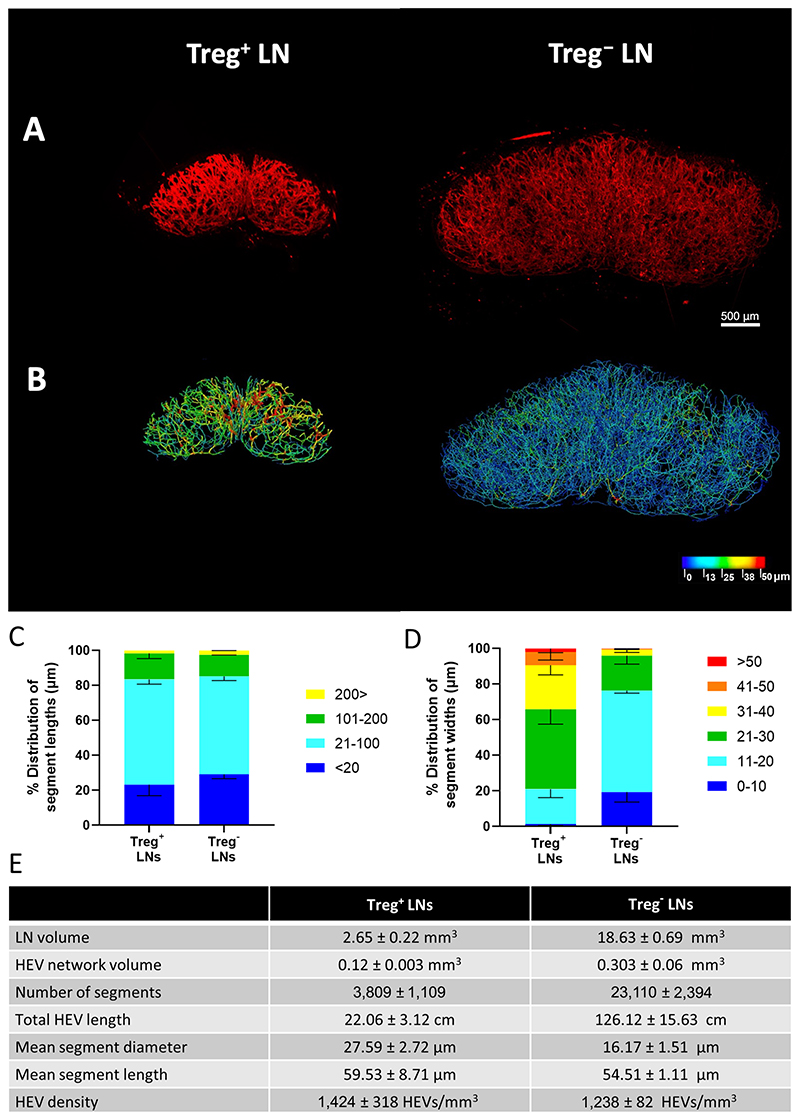
SPIM imaging and 3D analysis of LN HEV remodeling following Treg depletion. Whole LNs were labeled *in vivo* with 20 μg MECA-79 594, cleared using a 1:2 ratio of BABB and imaged by SPIM. **A,** Representative MIPs of a treatment-naïve inguinal LN (Treg^+^ LN, same as naïve LN in Fig. 1A) and a Treg-depleted inguinal LN (Treg^−^ LN). **B,** Complementary 3D rendered models of LN HEV networks derived using the vessel tools are color coded to represent the vessel widths. Relative distribution of HEV segment lengths (**C**) and widths (**D**) of pooled naïve inguinal LNs (Treg^+^ LNs, *n* = 3) and two Treg^−^ LNs (*n* = 2). Lengths and widths were divided into discrete bin (units shown are in μm) and the percentage of segments found in each bin was calculated and graphed. For pooled samples the mean is displayed with error bars representing SDs from the mean. **E,** Extracted numerical parameters of LN HEVs.

First, we examined the impact of Treg depletion on LN remodeling in tumor naïve Foxp3^DTR^ transgenic mice, treated six times with DT to maintain Treg depletion for approximately 3 weeks. Consistent with previous reports, Treg depletion led to lymphadenopathy as well as an increase in the extent of the overall LN HEV network (Supplementary Fig. S3; refs. 20, 50). A 3D imaging approach by LSFM revealed significant changes to the HEV network (Fig. 2A and B). A massive expansion (roughly 8-fold) in overall LN volume was observed after Treg depletion (Fig. 2E; refs. 20, 50). An approximately 9-fold expansion of the HEV network was also seen, characterized by an increase in the number of branching points as well as the total number of segments. These findings, which are in line with a previous study which used optical projection tomography (OPT; ref. 20) to measure HEV networks, suggests that the overall increase in HEV length is in part the result of vessel arborization leading to the formation of new vessels. A comparison of the vessel length distribution and vessel width distribution showed that the major difference between Treg-replete and Treg-depleted LNs was the distribution in vessel width where vessels with smaller widths of between 11 and 20 μm were more frequently observed after Treg depletion (Fig. 2C and D).

On the basis of two-dimensional (2D) imaging of LN sections, we previously reported a widening of LN HEV vessels as the major alteration driven by Treg depletion (18). However, the comprehensive 3D imaging and analysis described above clearly indicate that it is the formation of smaller width vessels which is the major alteration. This reiterates the notion that global statements to changes in vascular structures across entire tissue cannot be made with high precision based solely on 2D sectioning and imaging approaches (48).

### LN HEV Remodeling does not Distinguish Non-regressor and Regressor Tumors

**Figure 3:**
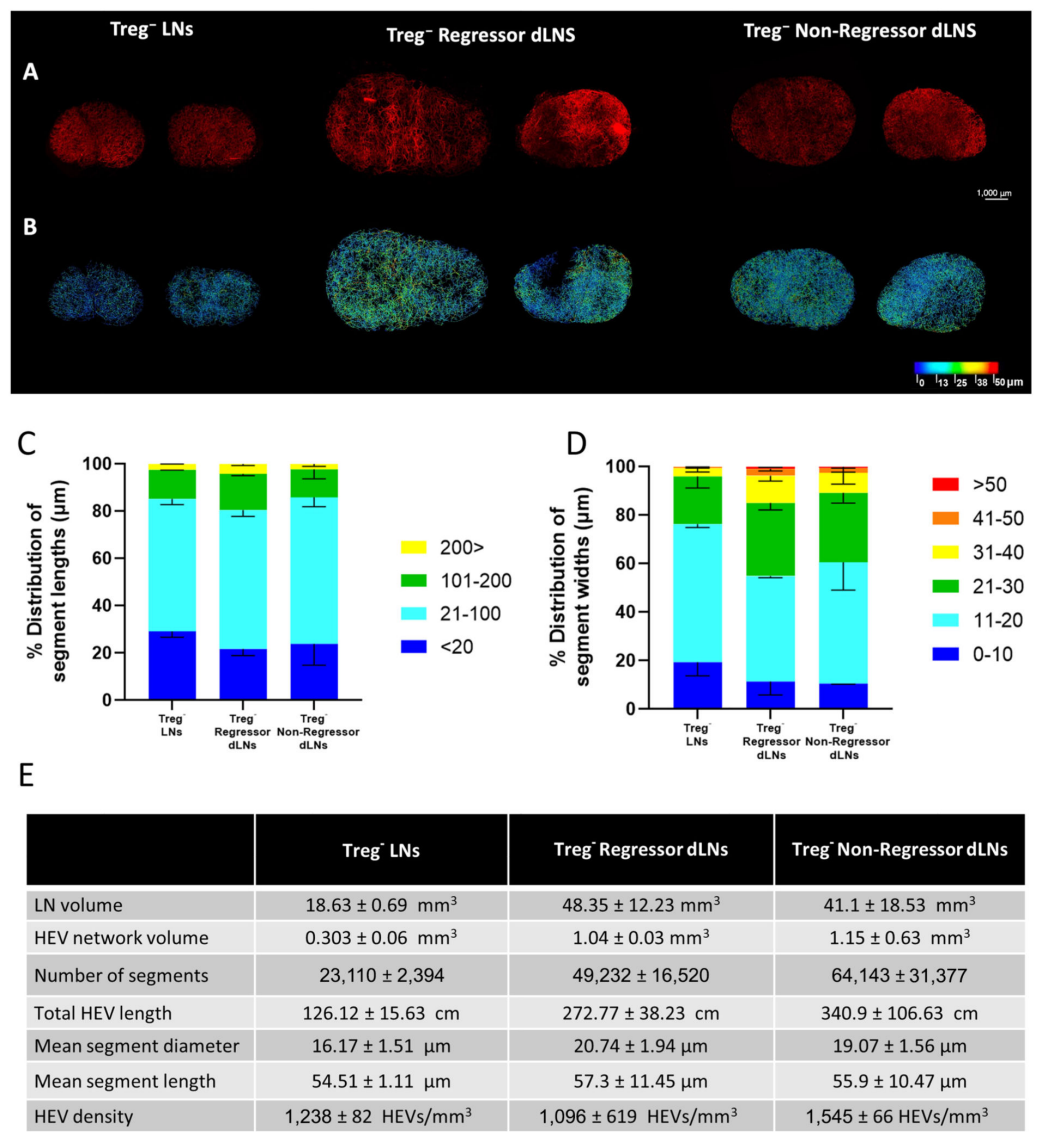
SPIM imaging and 3D analysis of LN HEVs following fibrosarcoma establishment and Treg depletion. Whole inguinal lymph nodes were labeled *in-vivo* with 20 μg MECA-79 594, cleared using a 1:2 ratio of BABB and imaged by SPIM. **A,** MIPs of Treg-depleted LNs (Treg^−^ LNs, *n* = 2), Treg-depleted non-regressor (Treg^−^ Non-Regressor dLNs, *n* = 2) and regressor dLNs (Treg^−^ Regressor dLNs, *n* = 2). **B,** Complementary 3D rendered models of LN HEV networks derived using the vessel tools are color coded to represent the vessel widths. Relative distribution of HEV segment lengths (**C**) and widths (**D**). Lengths and widths were divided into discrete bin (units shown are in μm) and the percentage of segments found in each bin was calculated and graphed. For pooled samples the mean is displayed with error bars representing standard deviations from the mean. **E,** Extracted numerical parameters of LN HEVs.

Given the extensive changes observed in response to either tumor (Fig. 1) or Treg depletion (Fig. 2), we next examined whether further alterations were associated with depleting Tregs in tumor-bearing mice. Upon development of a palpable tumor, Foxp3^DTR^ mice were treated with DT six times to deplete Tregs during the tumor growth phase. LN selected for HEV staining and imaging by LSFM were taken from mice bearing tumors which shrank following DT treatment (regressors) or from those where tumors continued to grow at similar rates to untreated mice (non-regressors; refs. 18, 19). We found that tumor establishment combined with Treg depletion led to a larger expansion in LN volume and overall extent of the HEV network as compared with Treg depletion alone (Fig. 3A). This was characterized by up to a 3-fold increase in LN volume and total HEV length (Fig. 3E). Both non-regressor and regressor dLNs from tumor-bearing mice had consistently larger segment numbers as compared with Treg-depleted LNs from non–tumor-bearing mice. We next examined whether any correlates of HEV remodeling were associated with the tumor response after Treg depletion. As shown in Fig. 3B, topological mapping of the dLN HEV network of regressor and non-regressors revealed the majority of HEVs to consist of small width vessels between 11 and 20 μm, as was also the case in LNs of Treg-replete, tumor-bearing mice (Fig. 1). Furthermore, the segment length and segment width distributions (Fig. 3C and D) were found to be largely comparable between non-regressor and regressor dLNs. These data indicate that dLN HEV networks are not differentially remodeled following successful Treg-targeted immune interventions.

As described in the Materials and Methods section, the vessels tools employed in this study rely on user-guided processing and manual adjusting of parameters to achieve appropriate segmentation (45). The processing of extensive LN HEV networks such as those seen in Treg-depleted LN HEVs (consisting of roughly 600 Z-slices) takes up to several months to appropriately segment. Future efforts to automize segmentation to a level similar to that achieved by human annotators will greatly increase the throughput of this analysis pipeline which at present is a limitation of this study.

### Treg Depletion Drives Development of HEV Networks in Some Tumors

**Figure 4:**
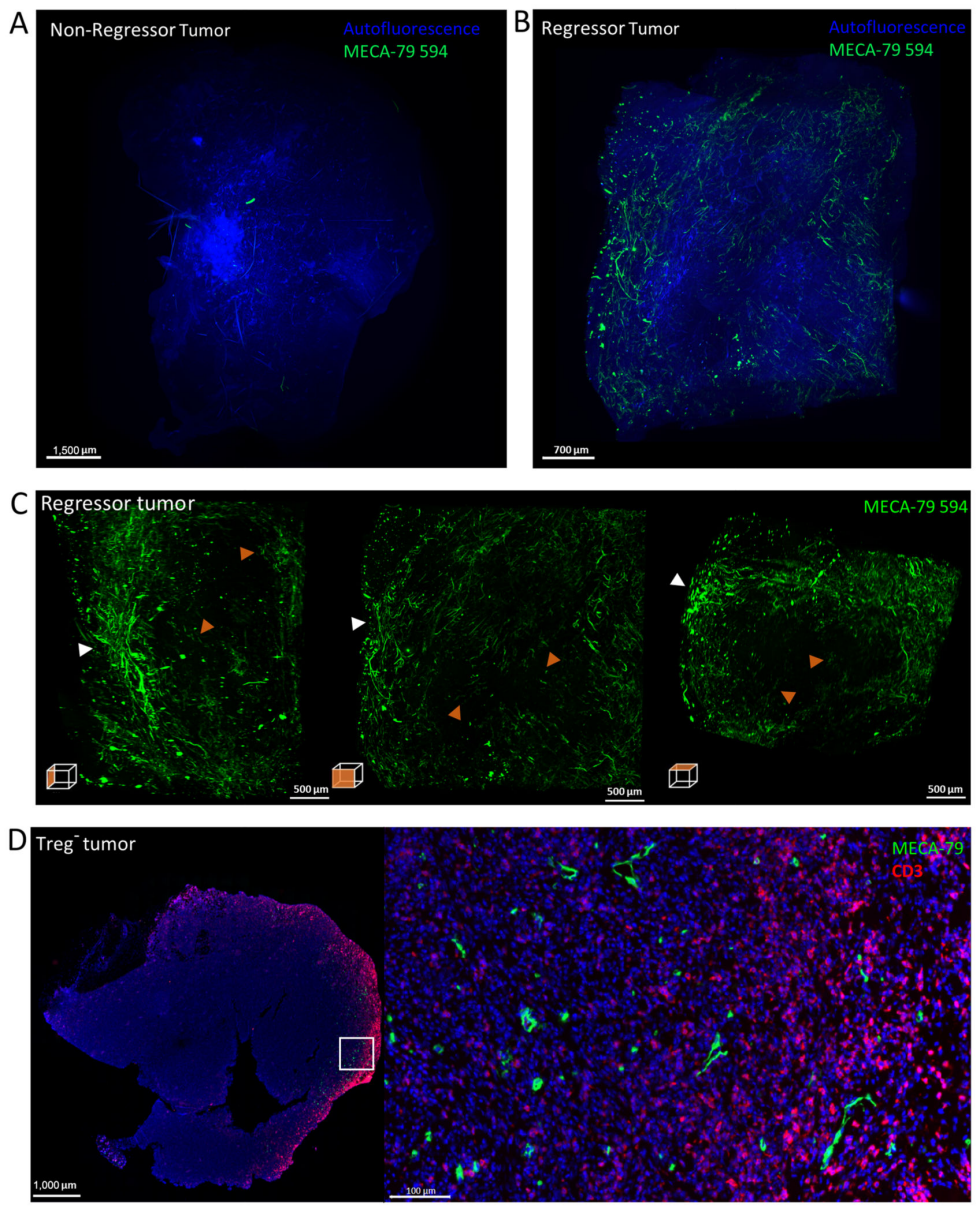
LSFM imaging of Treg-depleted fibrosarcomas. Representative images of MIPs of a non-regressor (*n* = 3, growth rate value of tumor shown: 0.07337 k,days^−1^ ; **A**) and regressor (*n* = 3, growth rate value of tumour shown: −0.04526 k,days^−1^; **B**) Treg-depleted fibrosarcoma *in vivo* labeled with 50 μg MECA-79 594 and imaged with the LaVision Ultramicroscope 2. **C,** MIPs of orthogonal views of regressor tumor. **D,** Paraffin-embedded Treg-depleted fibrosarcoma stained for HEVs (PNAd^+^; green) and T cells (CD3^+^; red). White arrowheads denote areas of dense PNAd^+^ clusters, orange arrowheads denote well-separated vessels that do not form extensive networks.

We next examined how HEV networks are organized and distributed across entire tumours; 3D LSFM imaging of entire *in vivo* labeled intratumoral HEV networks was conducted and a representative example of a Treg-depleted non-regressor and regressor tumor type is shown in Fig. 4.

3D imaging of a non-regressor Treg-depleted tumor did not reveal any extensive PNAd^+^ staining (Fig. 4A; Supplementary Fig. S4A). In contrast, an extensive network of PNAd^+^ vessels was readily observed in Treg-depleted regressor tumors (Fig. 4B; Supplementary Fig. S4B). Close inspection of the intratumoral HEV structure of the regressor tumor (Fig. 4C; Supplementary Fig. S4B) revealed a heterogenous global distribution pattern of PNAd^+^ vessels of varying density distinct from the organized well-defined hierarchy of branching venules seen in LNs. In tumors, dense clusters of PNAd^+^ vessels could be identified as well as several PNAd^+^ vessels that were well separated and not part of extensive PNAd^+^vessel networks. The majority of PNAd^+^ vessels were localized to the outermost edges of the tumor with the center of the tumor being largely devoid of any PNAd staining (Fig. 4B and C). Clear differences in the extent of the TA-HEV network between the regressor and non-regressor tumor confirm that intratumoral HEV formation is tightly linked to the control of tumor growth and colocalize with T cells as described previously (refs. 18, 19; Fig. 4D). To demonstrate that T-cell activation following Treg depletion influences intratumoral HEV neogenesis, Treg-depleted fibrosarcoma bearing mice were treated with anti-CD4 and anti-CD8 antibodies. Consistent with previous observations from 2D staining of tumor sections (18) LSFM imaging revealed that these tumors lacked HEVs (Supplementary Fig. S5A). Furthermore, no HEV were observed in MCA-induced fibrosarcomas from *Rag2*^−/−^ mice (Supplementary Fig. S5B). Collectively, the data described herein reveal that development of intratumoral HEV and not remodeling of the LN HEV network are the true correlate of antitumor immunity after depletion of Treg.

**Figure 5:**
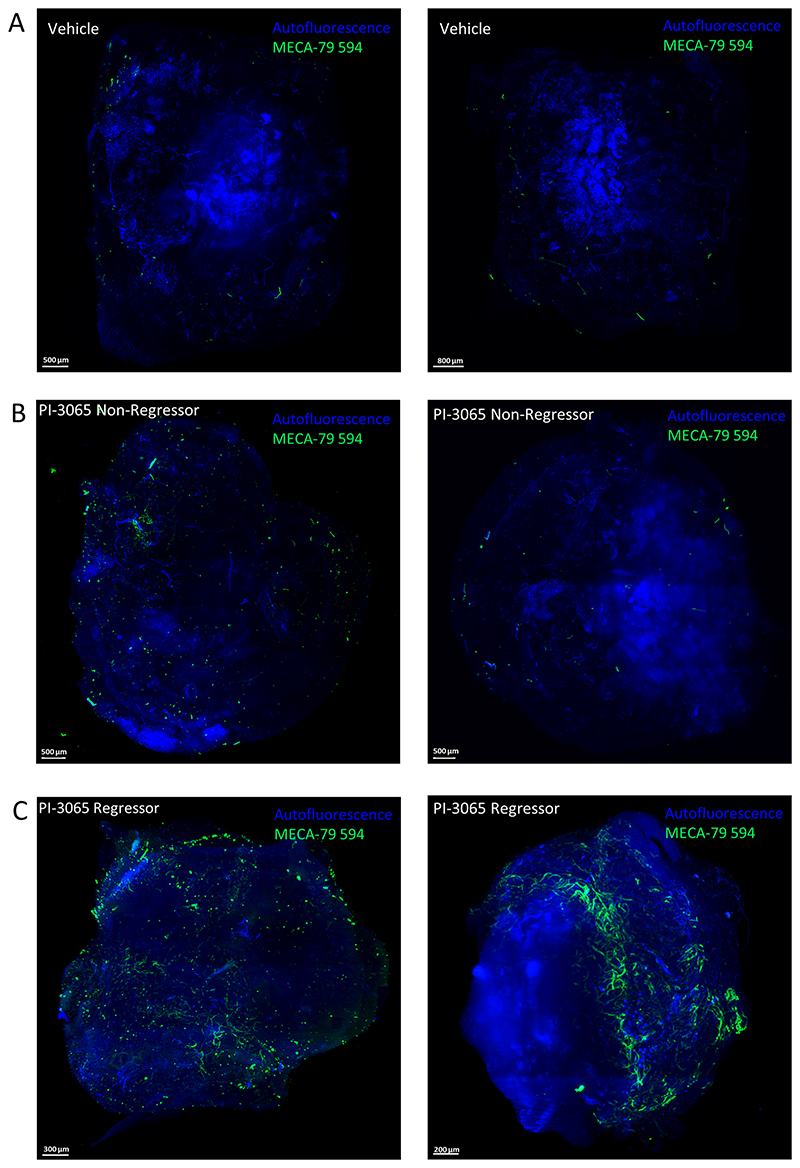
LSFM imaging of intratumoral HEVs in 4T1 tumors following PI-3065 treatment. MIPs of two vehicle-treated 4T1 tumors (*n* = 3; **A**), PI-3065–treated non-regressor tumor (*n* = 4; **B**), and PI-3065–treated regressor tumor (*n* = 2; **C**) *in vivo* labeled with 50 μg MECA-79 594 and imaged with the LaVision Ultramicroscope 2.

The link between Treg inactivation and intratumoral HEV neogenesis was subsequently confirmed in a separate tumor model using a PI3Kδ inhibitor (PI-3065) to inactivate Treg (52). As described previously, TA-HEVs are observed in 4T1 breast tumors of BALB/C mice treated with PI-3065 (39). HEV networks were compared in 4T1 tumors of mice which completely responded to treatment with PI-3065 (regressors) compared with those exhibiting a partial response (tumor growth slows down compared with untreated mice; we have termed these mice “non-regressors”; ref. 39). Using LSFM imaging, vehicle-treated tumors were found to lack HEVs (Fig. 5A) while PI-3065–treated regressor tumors were shown to have more extensive HEVs as compared with non-regressor tumors (Fig. 5B and C). Dense clusters of PNAd^+^vessels and several PNAd^+^vessels that were well separated were identified (Supplementary Fig. S6A and S6B) and these dense networks were found to be distributed across entire regressor (Supplementary Fig. S6B; Supplementary Movie S1 and S2) but not non-regressor tumors (Supplementary Fig. S6A; Supplementary Movie S3).

### Lymphotoxin β Receptor Agonist Antibodies Promote Intratumoural Development of HEV but do not Promote Tumor Rejection

Because tumor rejection in the fibrosarcoma model is associated with T-cell activation (induced by Treg depletion), we sought to determine whether development of TA-HEV in the absence of T-cell activation would be sufficient to initiate and control tumor growth, even in Treg-replete mice. Because signaling through the LTβR pathway has been implicated in the early development of SLOs (53) and importantly in the formation and maintenance of HEVs (23, 54–58), we set out to test our hypothesis by administering LTβR agonist antibodies to tumor-bearing mice.

**Figure 6:**
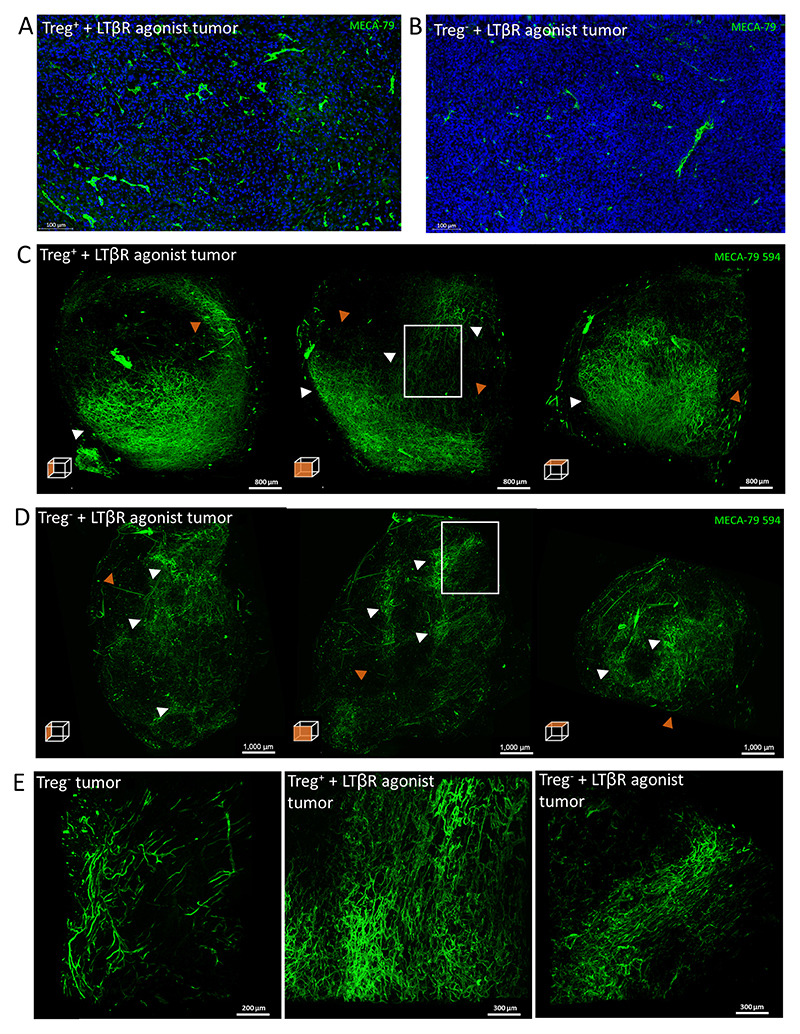
LSFM imaging of intratumoral HEVs following Treg depletion and LTβR agonist administration. Representative microscopy images of HEVs in a Treg^+^ + LTβR agonist tumor (**A**) and Treg^−^ + LTβR agonist tumor (**B**). HEVs were stained using the MECA-79 antibody. MIPs of orthogonal views of Treg-replete LTβR agonist tumor (**C**) and a Treg-depleted LTβR agonist tumor (**D**) *in vivo* labeled with 50 μg MECA-79 594 and imaged by the Lavision Ultramicroscope 2. **E,** Dense PNAd^+^ clusters of a Treg-depleted tumor (same as Fig. 4C, *n* = 3), Treg-replete LTBR agonist tumor (*n* = 1) and Treg-depleted LTβR agonist tumor (*n* = 2) (denoted by white box insert in **C** and **D**)were imaged at 2× magnification by LSMF. White arrowheads denote areas of dense PNAd^+^ clusters, orange arrowheads denote well-separated vessels that do not form extensive networks.

Administration of LTβR agonist resulted in the formation of PNAd^+^ vessels in 7 of 8 tumors from Treg-replete mice indicating that LTβR agonist treatment alone is indeed sufficient to drive HEV formation (see example in Fig. 6A). We also found that while Treg depletion normally results in intratumoural HEV development in around 30% to 50% of Treg-depleted mice, we saw intratumoural HEV in every mouse that was both Treg-depleted and treated with the LTβR agonist Abs (*n* = 7, see example in Fig. 6B). After LTβR agonist treatment both Treg-replete and -depleted tumors developed PNAd^+^vessels that were mostly localized to the outer regions of the whole tumor (Fig. 6C and D). While areas comprising of well-separated vessels that did not form extensive networks could be identified in tumors treated with LTβR agonist Abs, particularly dense clusters of PNAd^+^ vessels were often seen (Fig. 6C and D). Overall, this pattern, while similar to that observed in Treg-depleted tumors (compare with Fig. 4), implies that the LTβR agonist antibody promotes a more condensed HEV network. Indeed, examination of dense PNAd^+^ clusters at 2× magnification confirmed that the addition of LTβR agonist drives the formation of a densely packed HEV network which contrasts with the largely well-separated PNAd^+^ vessels seen in the Treg-depleted tumor without LTβR agonist (Fig. 6E).

**Figure 7:**
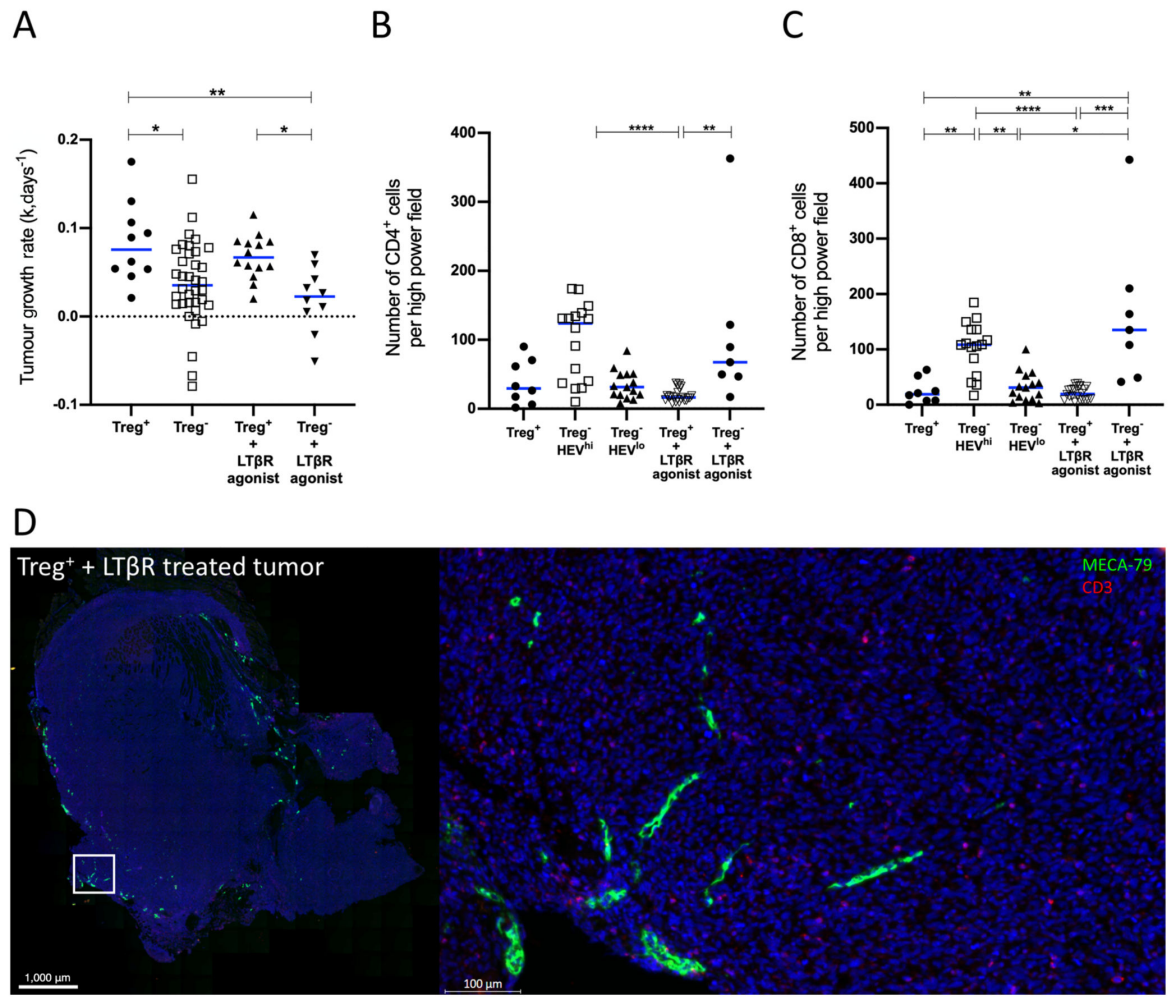
Impact of LTβR agonist Abs and Treg depletion on fibrosarcoma growth rate and extent of immune cell infiltration. **A,** Tumor growth rates (k, per day) for Treg^+^ (*n* = 10) Foxp3^DTR^ animals, Treg^−^ (*n*=36) Foxp3^DTR^ animals, Treg^+^ and LTβR agonist treated (*n*=14) Foxp3^DTR^ animals and Treg^−^ and LTβR agonist treated (*n* = 10) Foxp3^DTR^ animals. Statistical significance was determined by one-way ANOVA with Tukey multiple comparison test. (*, *P* <0.05; **, *P* <0.01). Number of CD4^+^ T cells (**B**) and CD8^+^ T cells (**C**) per high power field view for Treg^+^ (*n* = 8) Foxp3^DTR^animals, Treg^−^ HEV^hi^ (*n* = 16) Foxp3^DTR^ animals, Treg^−^ HEV^lo^ (*n* = 15), Treg^+^ and LTβR agonist treated (*n* = 22) Foxp3^DTR^ animals and Treg^−^ and LTβR agonist treated (*n* = 7) Foxp3^DTR^ animals. HEV^hi^ and HEV^lo^ tumours were distinguished by a threshold based on the median HEV area (threshold value 0.05809). Statistical significance was determined by Kruskal–Wallis test with Dunn multiple comparison test (*, *P* <0.05; **, *P* <0.01; ***, *P* <0.001; ****, *P* <0.0001). **D,** Paraffin-embedded Treg-replete fibrosarcoma treated with LTβR agonist was stained for HEVs (PNAd^+^; green) and T cells (CD3^+^; red).

Although imaging methods clearly revealed that HEV were induced by LTβR agonist Abs even when Treg were present, we found that this did not result in control of tumor growth (Fig. 7A). Corresponding to this finding, we found no increase in tumor-infiltrating lymphocyte number as a result of administering LTβR agonist Abs despite the presence of HEV (Fig. 7A and B). While administration of LTβR agonist Abs combined with Treg depletion resulted in development of HEV networks in all tumors, this was accompanied by only a very modest improvement in tumor control and T-cell infiltration (Fig. 7A–C). Indeed, in contrast to Treg-depleted mice, the intratumoral HEVs which formed following LTβR agonist treatment alone were not associated with a T cell infiltrate (Fig. 7D, compare with Fig. 4D). TA-HEVs in both Treg-depleted mice and those treated with LTβR agonist were not associated with a B-cell infiltrate (Supplementary Fig. S7). Collectively, these data indicate that in the context of MCA-induced fibrosarcomas, HEV induction in the absence of Treg depletion, is insufficient to promote an antitumor immune response.

In contrast, a recent report showed that tumors forming in mice inoculated with a fibrosarcoma cell line developed TA-HEVs surrounded by both CD8^+^ T cells and B cells with LTβR agonist antibodies alone. These tumors exhibited reduced weight compared with those from untreated mice, indicating a degree of tumor control (28). Such differential responses to LTβR agonist antibody treatment could be due to important differences in tumors which develop *in situ* versus those arising from inoculated tumor cell lines. Indeed, tumors derived from cells in culture have been shown to develop faster and to lack the architectural and cellular complexity that characterizes *in situ* tumors (59). Differences in vascularization may also be an important factor as it has been reported that tumors arising *in vivo* from injected cells lines are more angiogenic and comprise morphologically immature vessels that have been shown to be more sensitive to inflammatory cytokines such as TNF (60). For tumors that develop *in situ*, the evolving interactions between the immune system and cancer cells during the process of transformation (12) leads to a reduction in tumor immunogenicity and an immune activation threshold which must be overcome (by Treg depletion for example) before an effective antitumor immune response can take place (18). Tumor cells subjected to prolonged *in vitro* culture may conversely become more immunogenic due to a lack of immune selection pressure. Our study, which allowed TA-HEV development to be uncoupled from additional immune modulation clearly indicate that TA-HEV formation alone cannot facilitate tumor immunity without concomitantly targeting T cells. Overall, this is similar to the findings of most other studies, even those which rely on use of tumor cell lines. Successful treatment of tumors by delivery of LTβR agonist Abs (61) or recombinant LIGHT (which can also signal through LTβR; refs. 62–65), required concomitant T-cell activation *via* checkpoint blockade. Thus, most studies to date show that induction of PNAd^+^ vessels alone is not sufficient to lead to an increase in TIL numbers and control of tumor growth (61–65).

## Discussion

The ability to comprehensively map heterogeneity in any given tumor is often hindered by limited sampling and interobserver variability which arises from staining and analyzing thin tumor tissue sections. The ability to obtain a comprehensive global overview of microstructures in whole tissues deemed too large for conventional microscopy has however been made possible with developments in tissue clearing as well as the use of mesoscopic imaging techniques such as OPT and LSFM (48) and reviewed in ref. 66. Indeed, LSFM imaging has demonstrated substantial heterogeneity of stained markers across different tumor regions in diverse human solid tumors (37) with certain structural features measured in 3D correlating better with tumor staging than standard 2D staining parameters (37). 3D imaging has also been used to profile the immune compartment of both preclinical and human tumors, further dissecting the involvement of immune cells in tumor evasion and progression (34–36). Developments in whole mouse labeling and clearing protocols such as vDISCO (67) have allowed for the unbiased assessment of metastatic burden and immunotherapeutic drug delivery in several preclinical mouse models of cancer (68). Using *in vivo* fluorescent labeling combined with an autofluorescence reducing bleaching protocol and organic solvent-based clearing method we were able to resolve entire HEV networks found in whole fibrosarcomas and LNs. Furthermore, the refinement of existing computational tools allowed for the topological mapping of HEVs across whole LNs and facilitated quantification of global changes to the HEV network following tumor establishment and immune intervention.

Using these methods, we observed extensive HEV remodeling in Treg-depleted LNs, which aligns with and likely supports the massive expansion in lymphocyte number and the well-described lymphadenopathy seen after Treg depletion. These findings, which confirm previous data, validate our imaging approach while also supporting the premise that Tregs serve to maintain immune tolerance by restraining LN activation (69). Extensive HEV remodeling was also observed in dLN, and to a lesser extent ndLNs in response to tumor development. We postulate that these alterations to the LN HEV network may reflect early immunosurveillance mechanisms and an attempt to generate an antitumor immune response. Given that we have previously reported that the proportion of Treg is significantly increased in LNs of mice bearing carcinogen-induced fibrosarcomas (29), it is reasonable to speculate that immunosurveillance is thwarted, at least partly, by this enrichment of LN Treg. Indeed, Treg depletion unleashes antitumor immune responses in a significant proportion of animals. We did not however observe a pattern of LN HEV remodeling which specifically associated with a successful outcome to Treg depletion (i.e., tumor regression).

3D imaging of tumors showed a clear dichotomy in development of intratumoural HEV networks after Treg depletion; dense TA-HEV networks were found only in regressor and not non-regressor tumors. We have previously demonstrated that development of these HEV is driven by activated T cells, dependent on production of LT-α and TNF (18). Herein, we further show that induction of HEV in the absence of Treg depletion (induced by LTβR agonist Abs) does not promote T-cell infiltration and control of tumor growth. Collectively, our studies, and those of others using adoptively transferred T cells and/or PD-1 blockade, indicate that tumors successfully treated with immunotherapy contain HEV (28, 62–64, 70–72). Importantly, in each of these studies, HEV development is associated with T-cell activation and the distribution of HEV within a given tumor colocalizes with T cells. In this article, we also present findings using the 4T1 breast cancer model where Tregs are inactivated by pharmacologic targeting PI3Kδ (52). 3D imaging clearly shows that TA-HEV development is enhanced in treated mice which, as described previously, is accompanied by a significant increase in antigen-specific T cells (39). Overall, these findings imply a direct relationship whereby T-cell activation acts as a trigger for HEV development which subsequently serves to amplify the immune response by facilitating extravasation of T cells into the tumor mass. Findings from other studies indicate that TA-HEV support infiltration of naïve T cells into tumors (71, 73, 74). While Treg depletion is clearly crucial to the increased frequency of TIL observed in the MCA-induced tumor model described herein, we do not yet know whether a consequence of removing their immunosuppressive effects is enhanced due to priming of naïve T cells entering tumors as a result of TA-HEV development. We will seek to address this in our future studies.

Single-cell sequencing of high ECs from homeostatic, inflamed, or dedifferentiated LNs indicate that HEV exhibit a significant degree of plasticity, with clear implications for structural and functional heterogeneity within HEV networks (75). Thus, it is becoming increasingly clear that HEV can exhibit phenotypic heterogeneity with implications for lymphocyte recruitment function (75, 76). It is possible that the intratumoral PNAd^+^ vessels induced by administration of LTβR agonist Abs are distinct from those induced by Treg depletion as their presence is not linked to enhanced T-cell infiltration and control of tumor growth. As mentioned above, this is in contrast to a recent study which showed LTβR agonist treatment alone to be sufficient in inducing TA-HEVs which were associated with the presence of T and B cells as well as a reduction in tumor weight (28). Tumors that develop *in situ* may therefore respond differently to LTβR agonist treatment.

While the findings of this study indicate clear qualitative differences in TA-HEV density following either Treg and/or LTβR targeted therapy, future work should seek to derive quantitative descriptors of TA-HEVs as we have achieved for LN HEV networks. Current vessel tools, which rely on user-guided processing and manual adjusting of parameters to achieve appropriate segmentation (45), do not allow for the processing of extensive TA-HEV networks. Recently, machine learning approaches for automatic segmentation have been applied to whole brain vascular networks imaged by LSFM (77). In this work, automatic segmentation was found to be of similar quality to that achieved by human annotators (77). In the future, a similar approach could provide useful for the processing of larger tumor HEV datasets especially given that image segmentation is the rate-limiting step of this computational pipeline. Indeed, with direct relevance to cancer research, deep learning–based analysis has significantly shortened tasks (such as the quantification of individual micrometastases in whole mice) to only a few days which would otherwise take humans several months to years to achieve (67).

Overall our data point to a compelling link between T-cell activation and induction of “useful” HEV, that is, PNAd^+^vessels which serve to amplify the antitumor T-cell response and contribute to control of tumor growth (18, 19). This leads us to speculate that TA-HEV also exhibit heterogeneity and that specific drivers of vessel development influence their phenotype and function. Thus, it is clearly important to further interrogate mechanisms underpinning interactions between TA-HEV–like vessels and a “successful” T-cell response. Such approaches are essential to identify routes for inducing useful HEV in tumors as well as new targets for enhancing the efficacy of cancer-specific T cells.

## Supplementary Material

Supplementary Material

Supplementary Movie 1

Supplementary Movie 2

Supplementary Movie 3

## Data Availability

The computational tools used in this study are accessible on GITHUB (https://github.com/gibbogle/vessel-tools.git). All 3D datasets generated in this study are available upon request from the corresponding author.

## Significance

We used three-dimensional imaging methods with computational tools to analyze networks of specialized blood vessels called HEVs in LNs and tumors. By applying these techniques in a mouse model of carcinogen-induced tumors, we could identify network changes after depletion of Tregs.
