# Supplementary figures and images for: Three-dimensional Imaging Reveals Immune-driven Tumor-associated High Endothelial Venules as a Key Correlate of Tumor Rejection Following Depletion of Regulatory T Cells
Source: Cancer Res Commun. 2022 Dec 15;2(12):1641–56. doi: 10.1158/2767-9764.CRC-21-0123 (PMC7614106; doi:10.1158/2767-9764.CRC-21-0123)

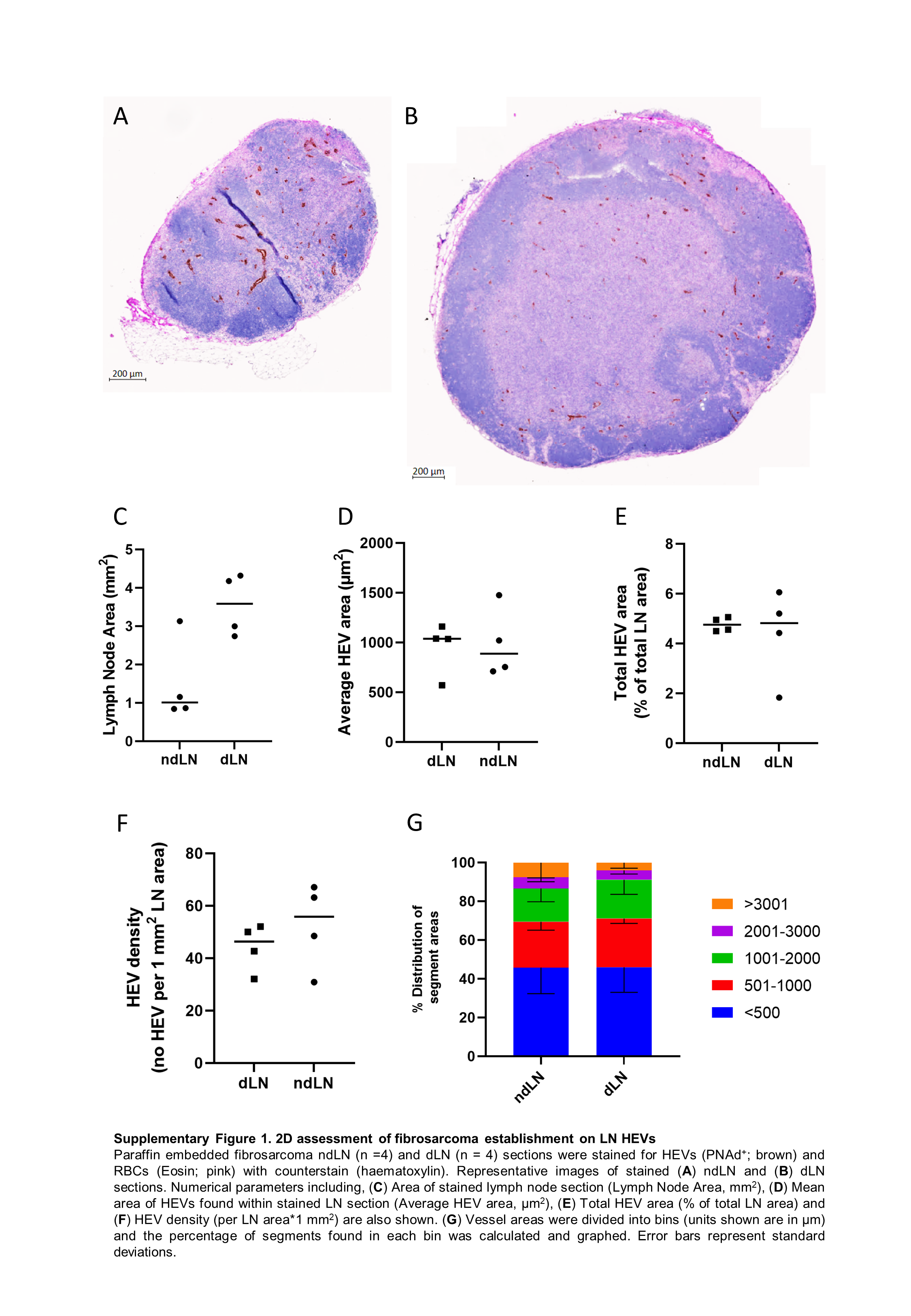

Supplement: Figure S1 — 2D assessment of fibrosarcoma establishment on LN HEVs [file crc-21-0123-s01.png]

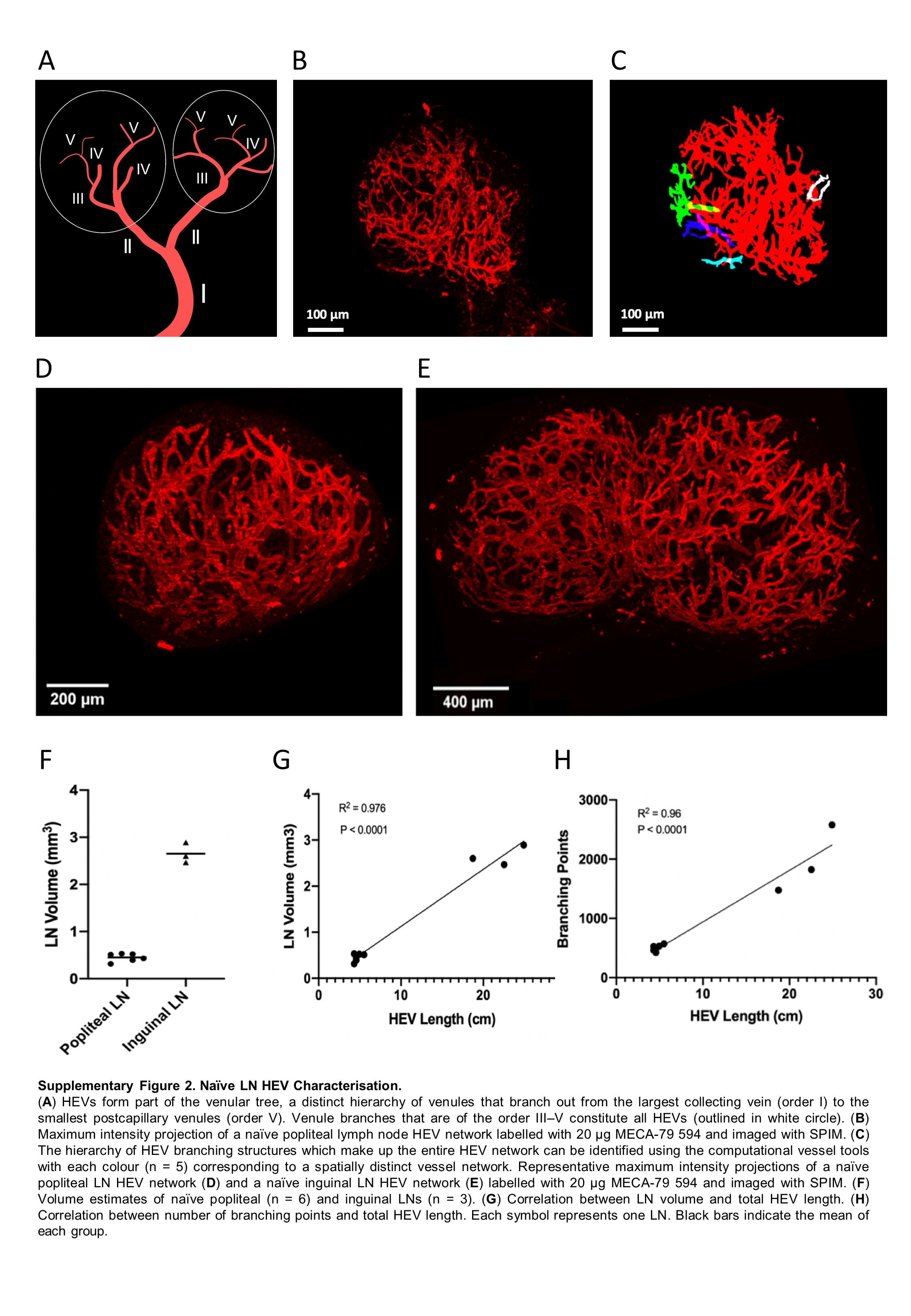

Supplement: Figure S2 — Naïve LN HEV Characterisation. [file crc-21-0123-s02.png]

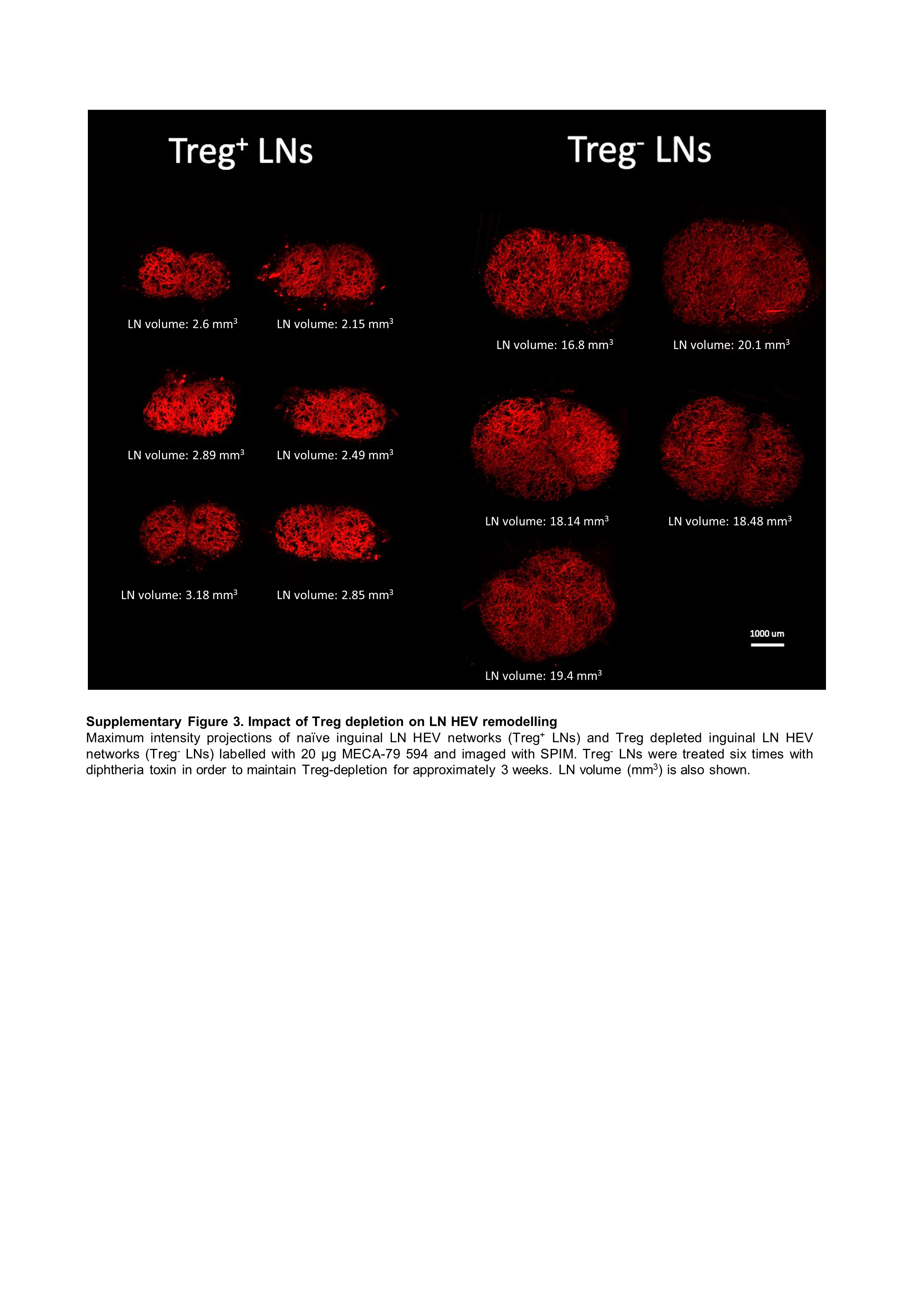

Supplement: Figure S3 — Impact of Treg depletion on LN HEV remodelling [file crc-21-0123-s03.png]

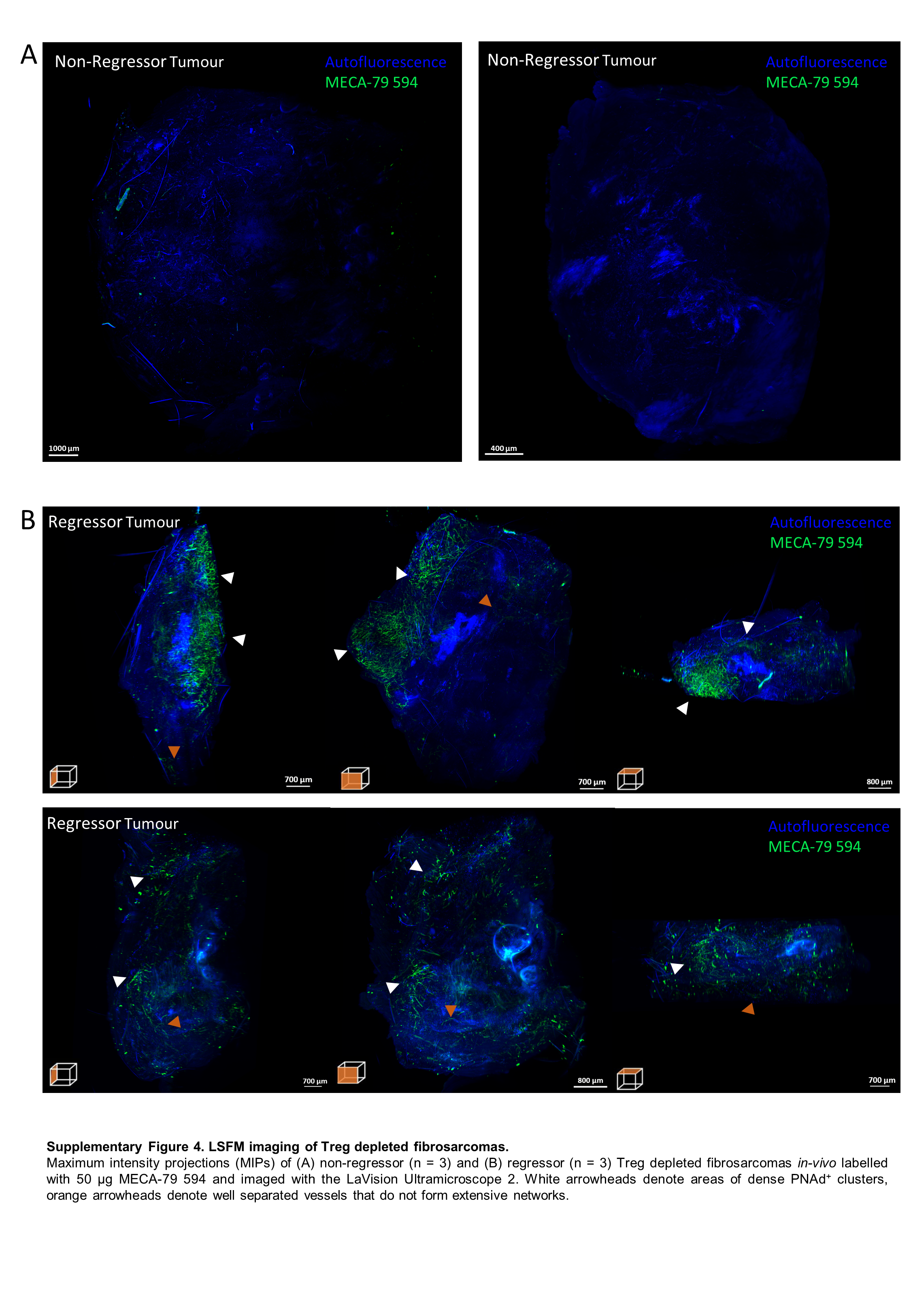

Supplement: Figure S4 — LSFM imaging of Treg depleted fibrosarcomas. [file crc-21-0123-s04.png]

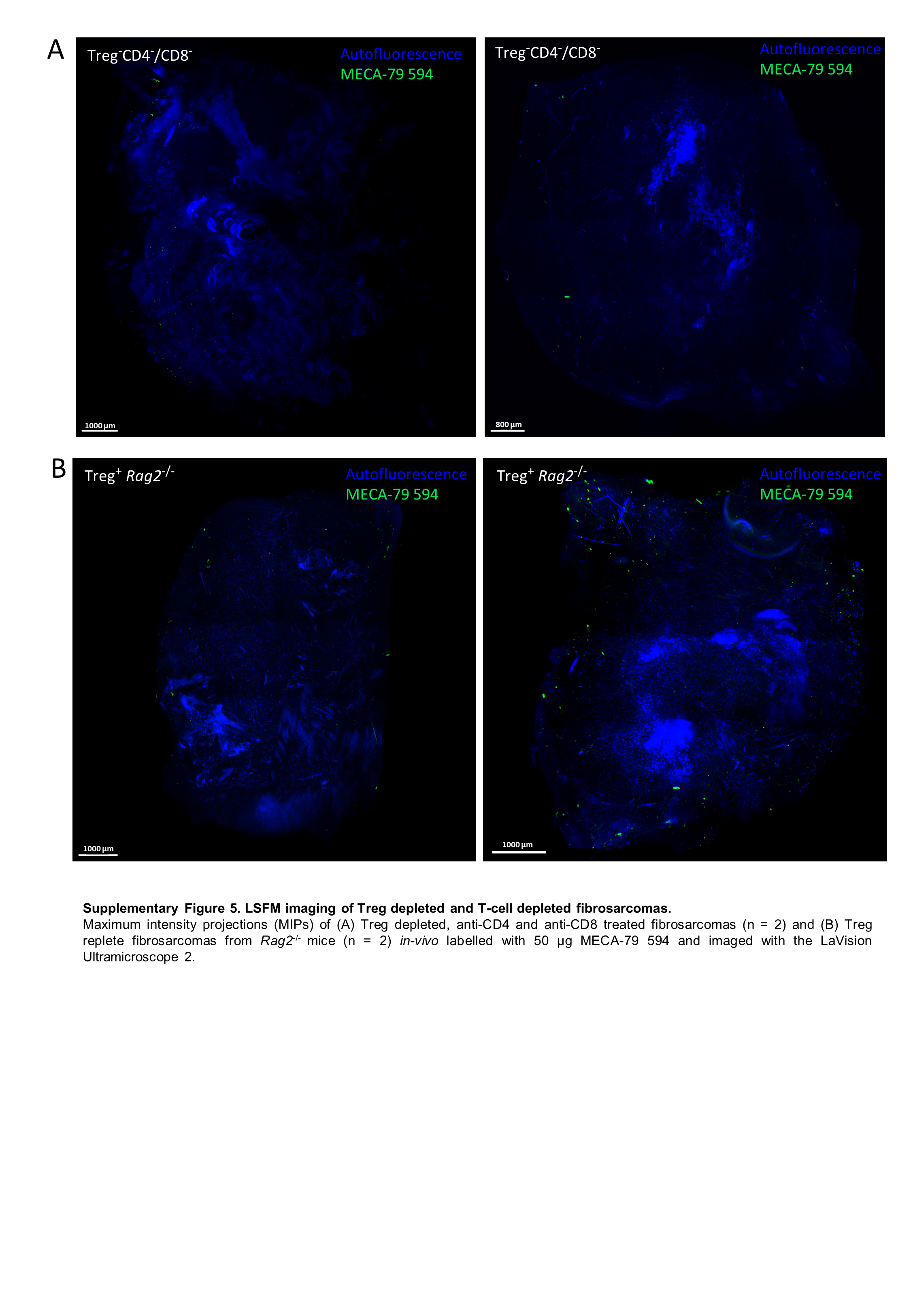

Supplement: Figure S5 — LSFM imaging of Treg depleted and T-cell depleted fibrosarcomas. [file crc-21-0123-s05.png]

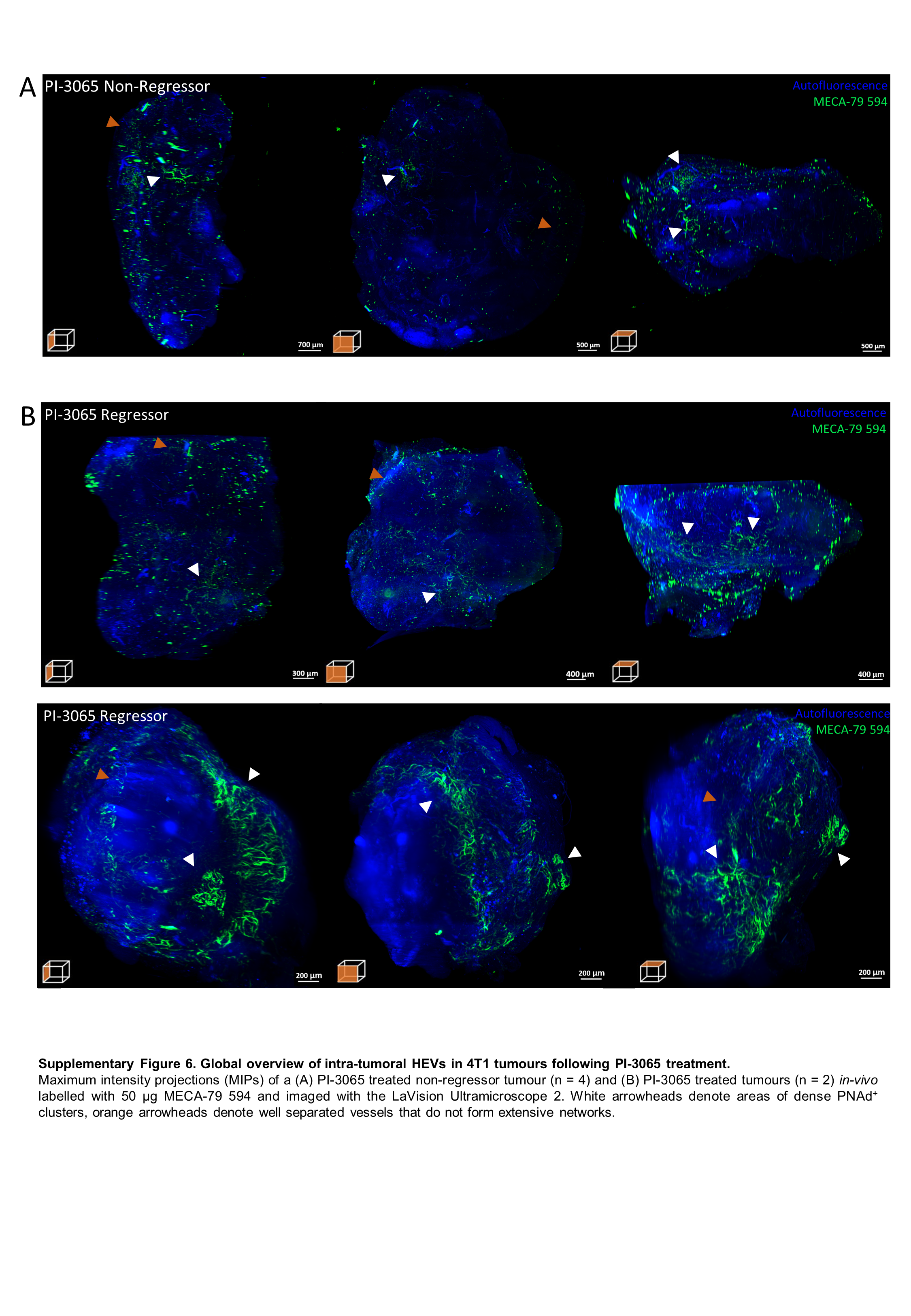

Supplement: Figure S6 — Global overview of intra-tumoral HEVs in 4T1 tumours following PI-3065 treatment. [file crc-21-0123-s06.png]

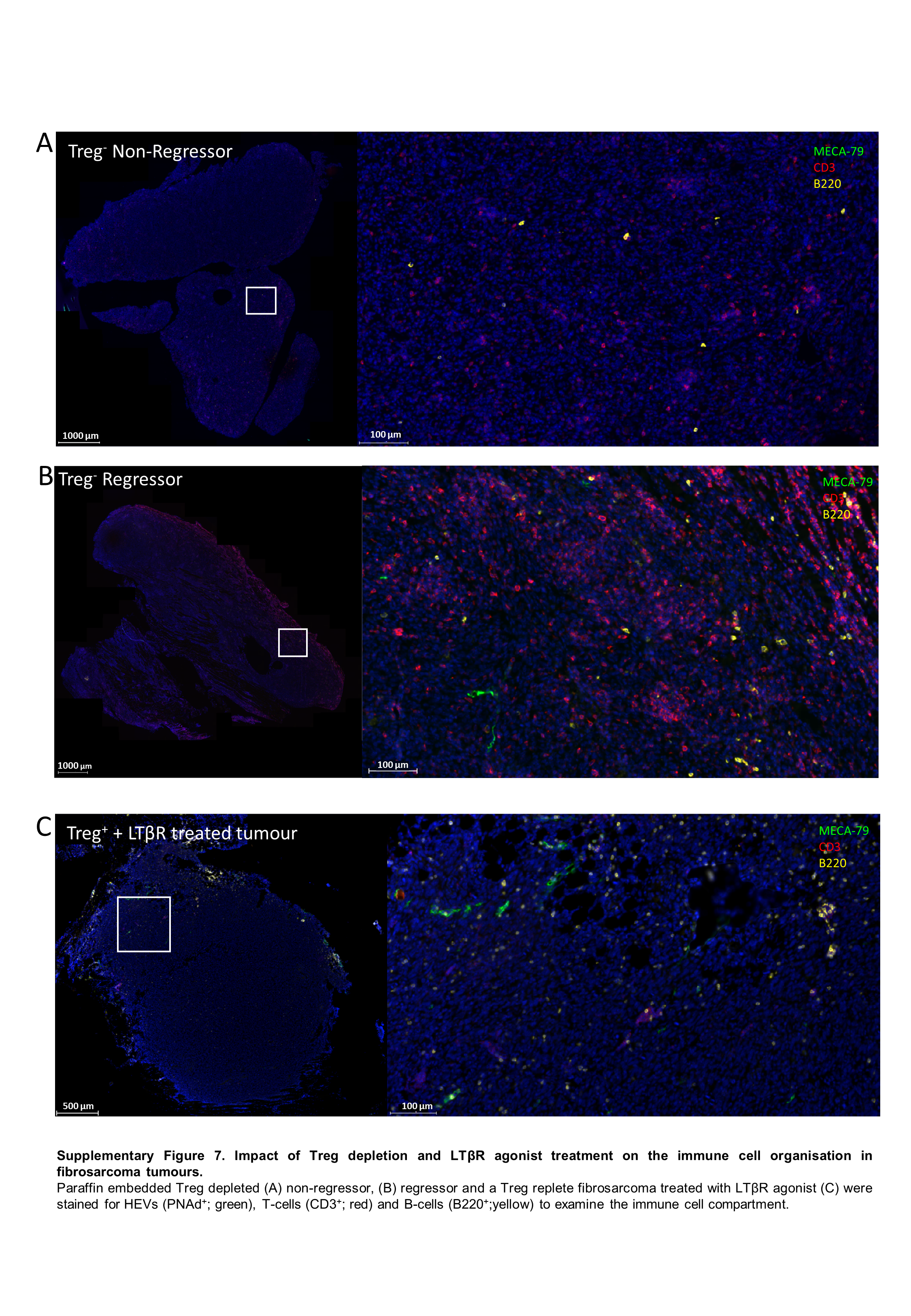

Supplement: Figure S7 — Impact of Treg depletion and LTβR agonist treatment on the immune cell organisation in fibrosarcoma tumours. [file crc-21-0123-s07.png]
