# Supplementary material for: Three-dimensional Imaging Reveals Immune-driven Tumor-associated High Endothelial Venules as a Key Correlate of Tumor Rejection Following Depletion of Regulatory T Cells
Source: Cancer Res Commun. Author manuscript; Available in PMC 2023 Jan 25. (PMC7614106; doi:10.1158/2767-9764.CRC-21-0123)
Supplement: Supplementary file 1 [file EMS159490-supplement-Supplementary_Material.pdf]

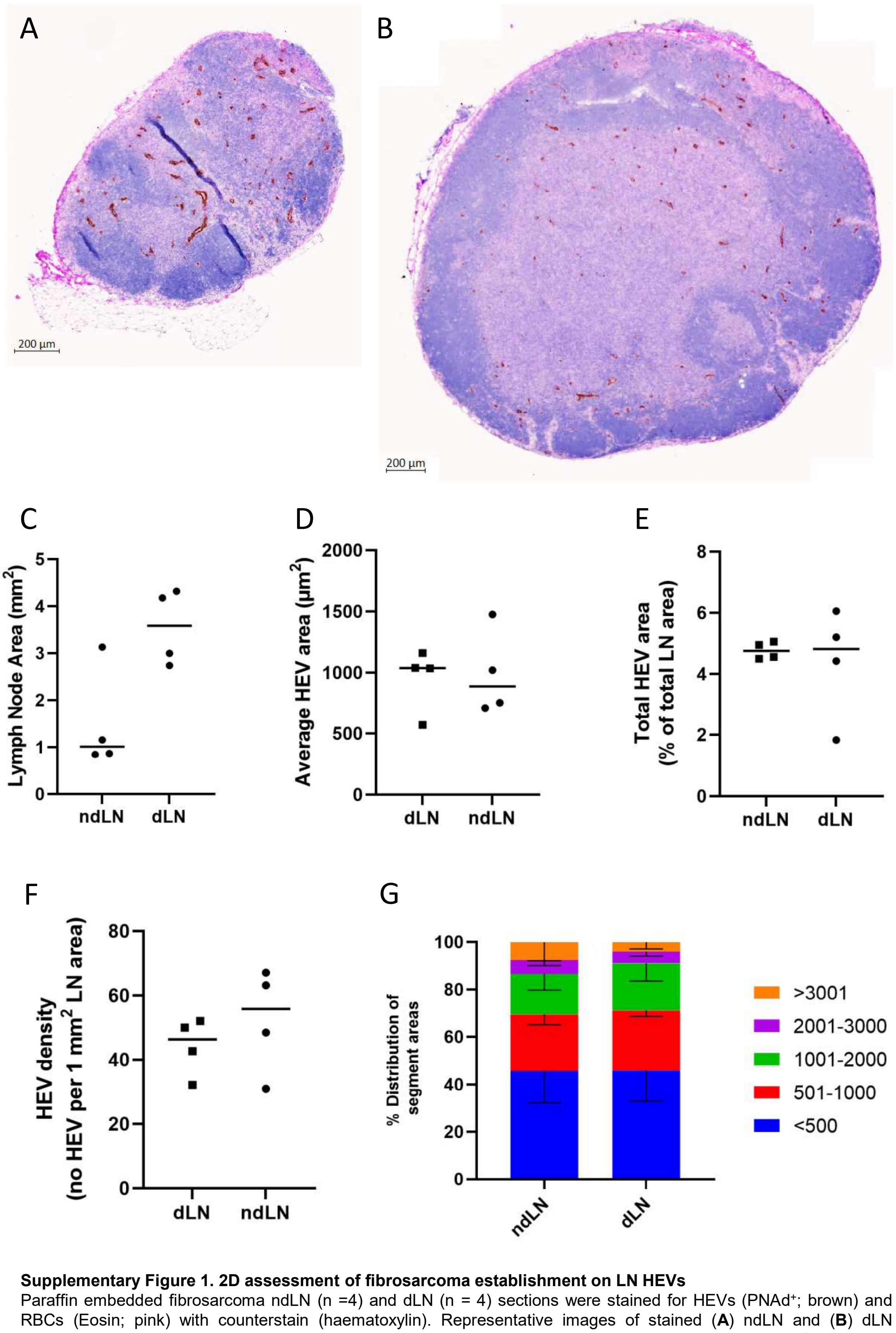

Supplementary Figure 1. 2D assessment of fibrosarcoma establishment on LN HEVs

Paraffin embedded fibrosarcoma ndLN (n =4) and dLN (n = 4) sections were stained for HEVs (PNAd$^+$; brown) and RBCs (Eosin; pink) with counterstain (haematoxylin). Representative images of stained (**A**) ndLN and (**B**) dLN sections. Numerical parameters including, (**C**) Area of stained lymph node section (Lymph Node Area, mm^2), (**D**) Mean area of HEVs found within stained LN section (Average HEV area, μm^2), (**E**) Total HEV area (% of total LN area) and (**F**) HEV density (per LN area*1 mm^2) are also shown. (**G**) Vessel areas were divided into bins (units shown are in µm) and the percentage of segments found in each bin was calculated and graphed. Error bars represent standard deviations.

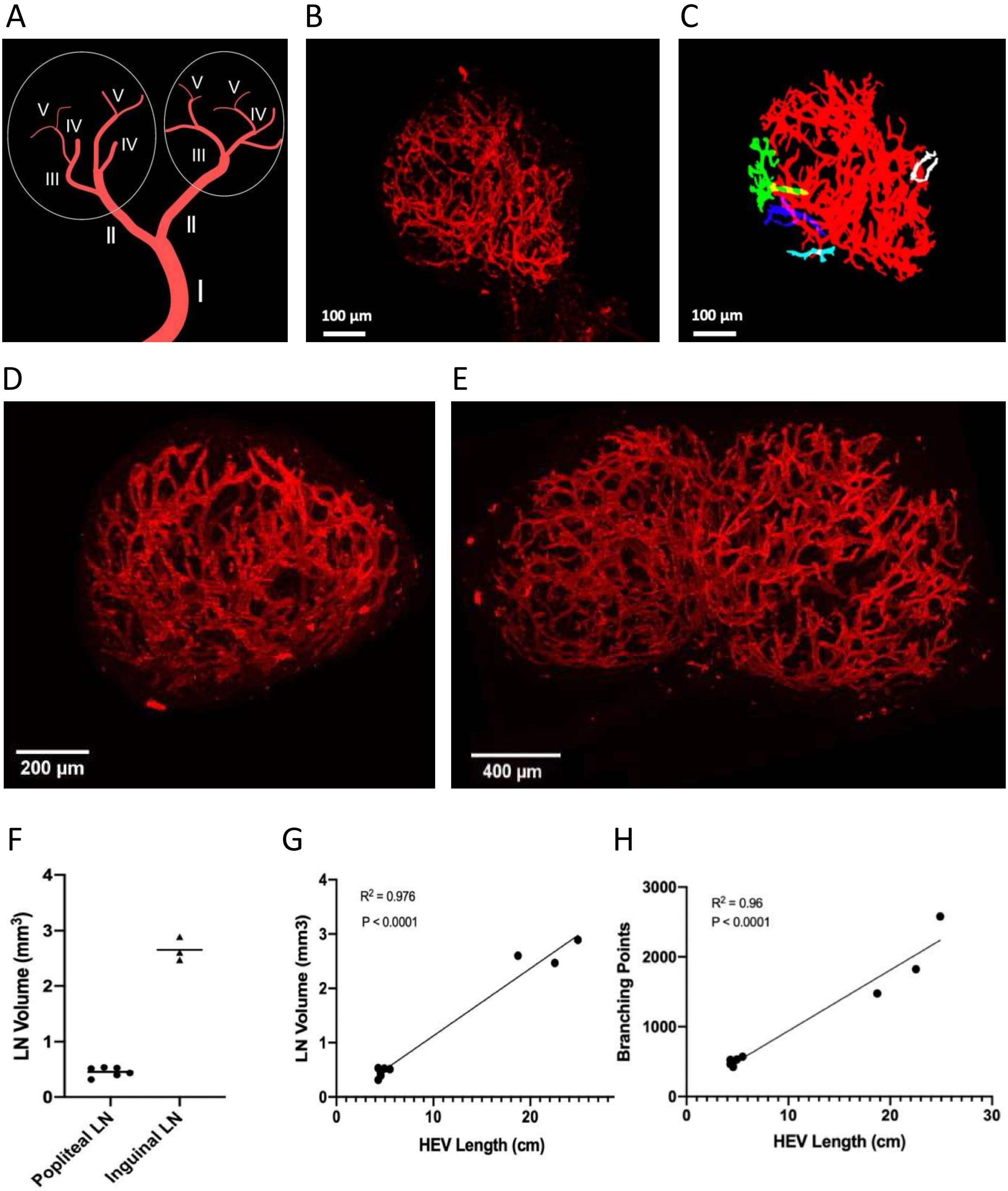

Supplementary Figure 2. Naïve LN HEV Characterisation.

(**A**) HEVs form part of the venular tree, a distinct hierarchy of venules that branch out from the largest collecting vein (order I) to the smallest postcapillary venules (order V). Venule branches that are of the order III–V constitute all HEVs (outlined in white circle). (**B**) Maximum intensity projection of a naïve popliteal lymph node HEV network labelled with 20 µg MECA-79 594 and imaged with SPIM. (**C**) The hierarchy of HEV branching structures which make up the entire HEV network can be identified using the computational vessel tools with each colour (n = 5) corresponding to a spatially distinct vessel network. Representative maximum intensity projections of a naïve popliteal LN HEV network (**D**) and a naïve inguinal LN HEV network (**E**) labelled with 20 µg MECA-79 594 and imaged with SPIM. (**F**) Volume estimates of naïve popliteal (n = 6) and inguinal LNs (n = 3). (**G**) Correlation between LN volume and total HEV length. (**H**) Correlation between number of branching points and total HEV length. Each symbol represents one LN. Black bars indicate the mean of each group.

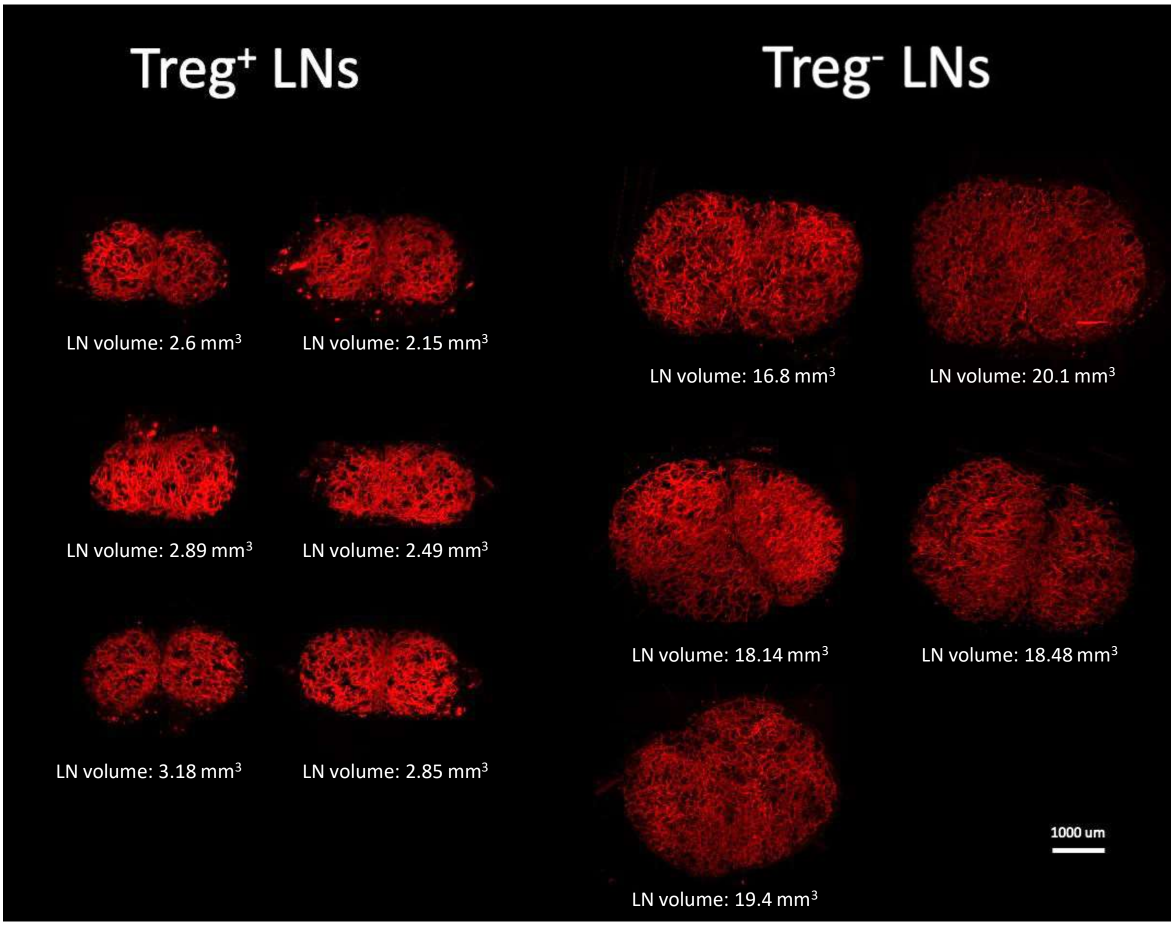

Supplementary Figure 3. Impact of Treg depletion on LN HEV remodelling
Maximum intensity projections of naïve inguinal LN HEV networks (Treg$^+$ LNs) and Treg depleted inguinal LN HEV networks (Treg$^-$ LNs) labelled with 20 µg MECA-79 594 and imaged with SPIM. Treg$^-$ LNs were treated six times with diphtheria toxin in order to maintain Treg-depletion for approximately 3 weeks. LN volume (mm^3) is also shown.

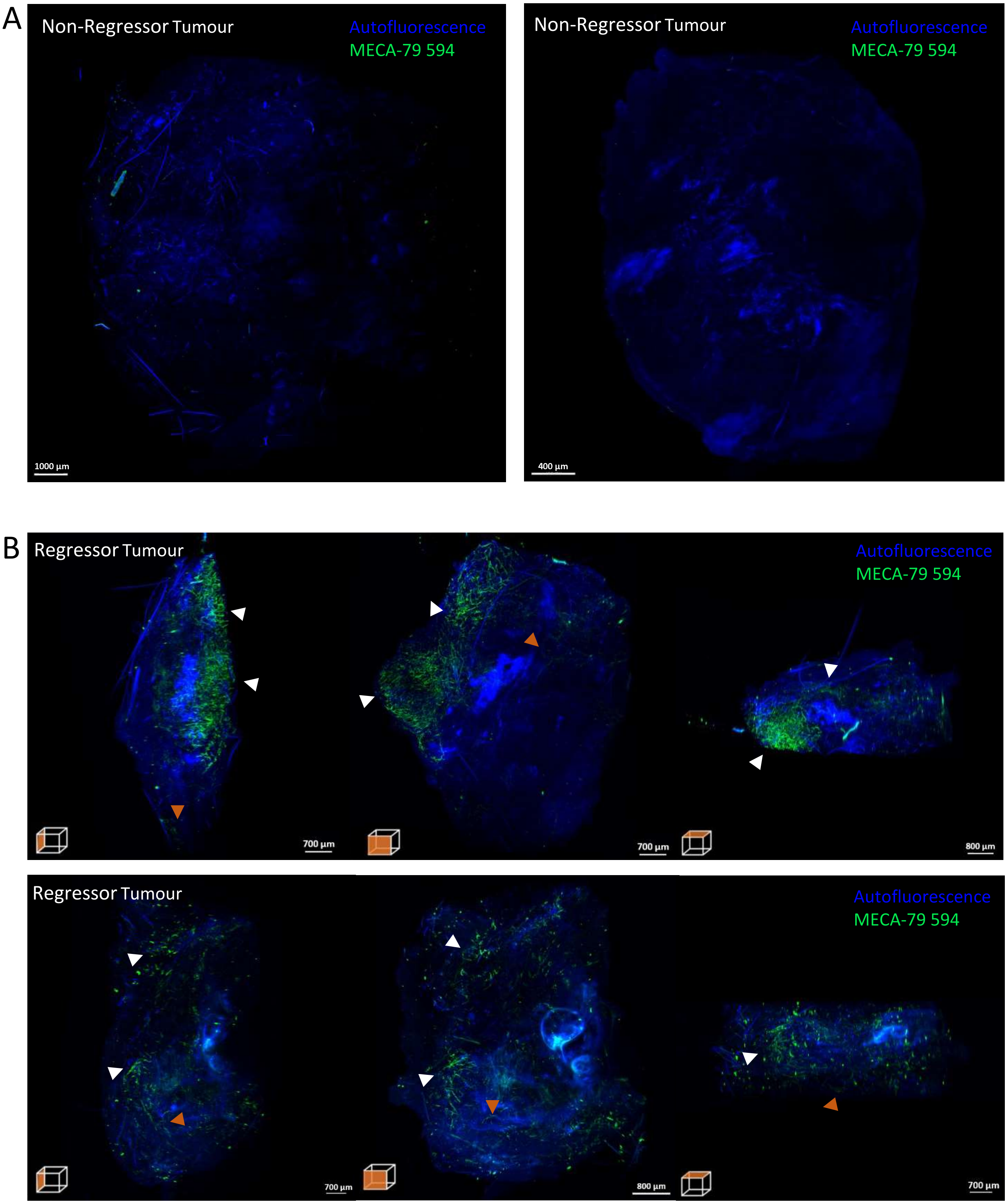

Supplementary Figure 4. LSFM imaging of Treg depleted fibrosarcomas.
Maximum intensity projections (MIPs) of (A) non-regressor (n = 3) and (B) regressor (n = 3) Treg depleted fibrosarcomas *in-vivo* labelled with 50 µg MECA-79 594 and imaged with the LaVision Ultramicroscope 2. White arrowheads denote areas of dense PNAd$^+$ clusters, orange arrowheads denote well separated vessels that do not form extensive networks.

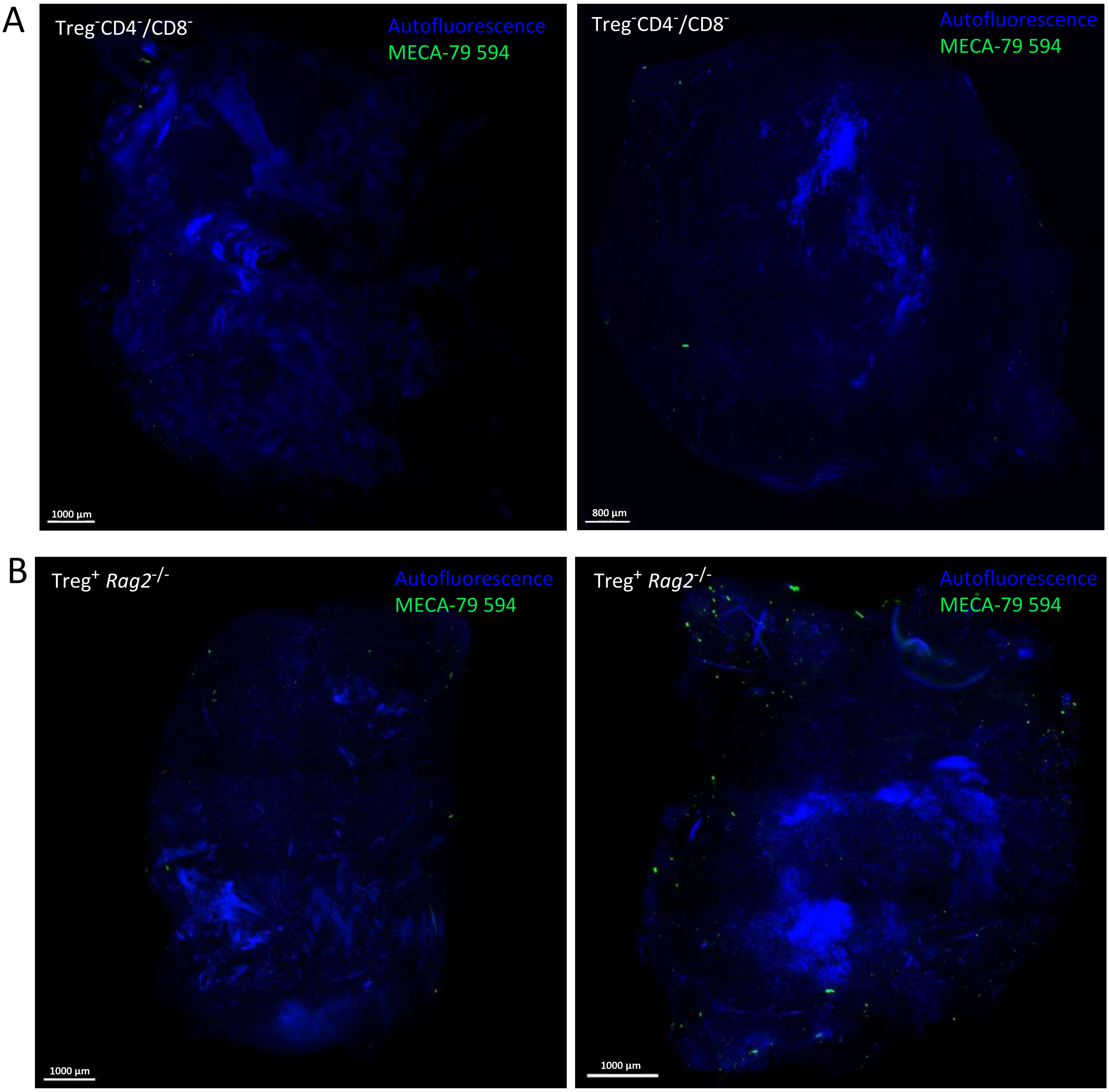

Supplementary Figure 5. LSFM imaging of Treg depleted and T-cell depleted fibrosarcomas.
Maximum intensity projections (MIPs) of (A) Treg depleted, anti-CD4 and anti-CD8 treated fibrosarcomas (n = 2) and (B) Treg replete fibrosarcomas from *Rag2*$^{-/-}$ mice (n = 2) *in-vivo* labelled with 50 µg MECA-79 594 and imaged with the LaVision Ultramicroscope 2.

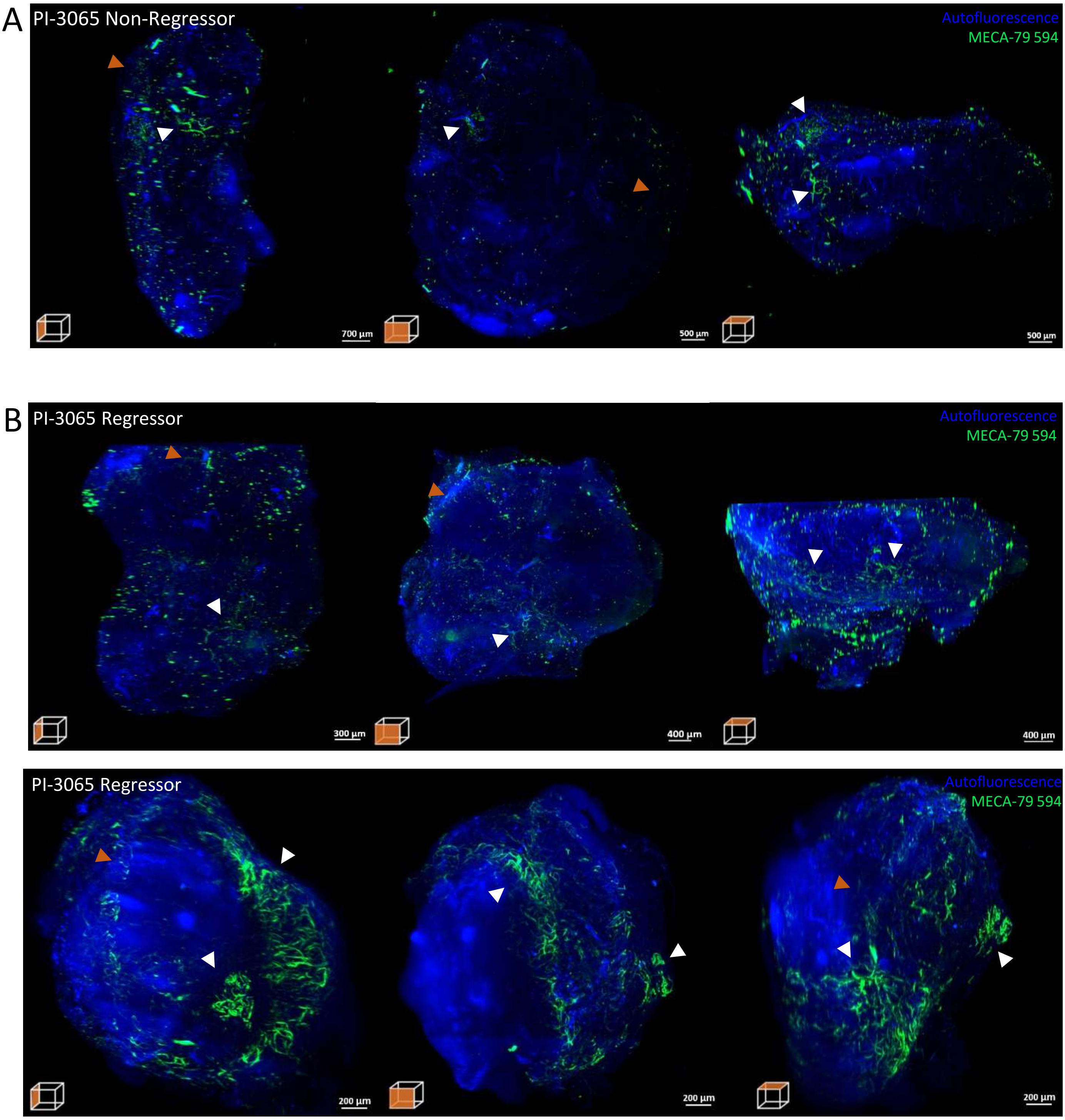

Supplementary Figure 6. Global overview of intra-tumoral HEVs in 4T1 tumours following PI-3065 treatment.
Maximum intensity projections (MIPs) of a (A) PI-3065 treated non-regressor tumour (n = 4) and (B) PI-3065 treated tumours (n = 2) *in-vivo* labelled with 50 µg MECA-79 594 and imaged with the LaVision Ultramicroscope 2. White arrowheads denote areas of dense PNAd^{+} clusters, orange arrowheads denote well separated vessels that do not form extensive networks.

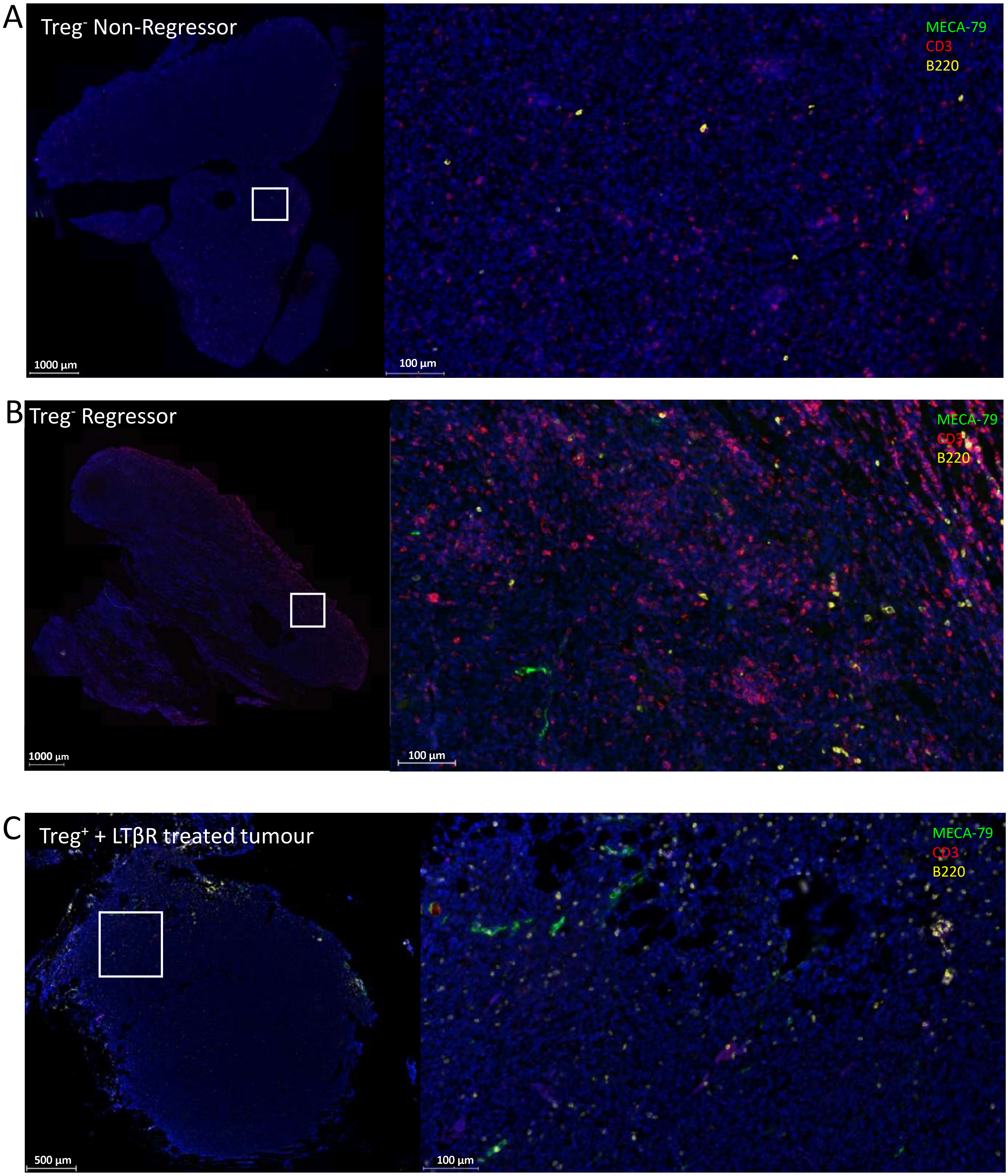

Supplementary Figure 7. Impact of Treg depletion and LTβR agonist treatment on the immune cell organisation in fibrosarcoma tumours.

Paraffin embedded Treg depleted (A) non-regressor, (B) regressor and a Treg replete fibrosarcoma treated with LTβR agonist (C) were stained for HEVs (PNAd$^+$; green), T-cells (CD3$^+$; red) and B-cells (B220$^+$;yellow) to examine the immune cell compartment.
